# Mapping the spatial heterogeneity of global land use and land cover from 2020 to 2100 at a 1 km resolution

**DOI:** 10.1038/s41597-023-02637-7

**Published:** 2023-10-28

**Authors:** Tianyuan Zhang, Changxiu Cheng, Xudong Wu

**Affiliations:** 1 State Key Laboratory of Earth Surface Processes and Resource Ecology, Beijing Normal University, Beijing, 100875 PR China; 2 National Tibetan Plateau Data Center, Beijing, 100101 PR China; 3 School of Soil and Water Conservation, Beijing Forestry University, Beijing, 100083 PR China; 4 Research Department of Complexity Science, Potsdam Institute for Climate Impact Research, Potsdam, 14473 Germany

**Keywords:** Environmental sciences, Climate sciences

## Abstract

A fine global future land use/land cover (LULC) is critical for demonstrating the geographic heterogeneity of earth system dynamics and human-earth interaction. In this study, we produced a 1 km global future LULC dataset that takes into account future climate and socio-economic changes as well as the impact of simulated results of the former year on temporally adjacent periods. By incorporating the variations in climatic and socio-economic factors, we differentiated LULC suitability probabilities for historical and future periods across representative SSP-RCP scenarios. Then, by using an improved cellular automata model-PLUS to simulate the patch-level changes of various land classes, we iteratively downscaled water-basin-level LULC demands in various future scenarios to a spatial resolution of 1 km. Our dataset achieves a high degree of simulation accuracy (Kappa = 0.94, OA = 0.97, FoM = 0.10) and precisely captures the spatial-temporal heterogeneity of global LULC changes under the combined effects of climate change and socio-economic development. This robust and fine-scale LULC dataset provides valuable spatially-explicit information essential for earth system modeling and intricate dynamics between anthropogenic activities and the environment.

## Background & Summary

Land use and land cover (LULC) change reflects the intricate interaction between climate change and intensive human activities^1^ and is closely correlated to various terrestrial processes such as biodiversity, earth surface energy balance, atmospheric circulation, and carbon cycle^2^. From 1982 to 2016, 60% of land transformation was due to anthropogenic activities such as the invasion of cropland and built-up area^3^. This trend may continue to intensify in the foreseeable future with the ongoing population growth and economic development. Predicting global-scale land use dynamics under future socio-economic and climate scenarios is essential for implementing effective land utilization decisions towards sustainable development goals. Furthermore, global LULC at a suitable geospatial resolution serves as a key input of the earth system model, which represents a crucial component for simulating the geographic heterogeneity of earth system dynamics as well as anthropogenic environmental impacts.

Early LULC projections mostly forecast global land use demands at subregional levels, which are generally obtained from integrated assessment models (IAMs) such as AIM (Asia‐Pacific integrated model), modular applied general equilibrium tool (MAGNET), Integrated Model to Assess the Global Environment (IMAGE), and Global Change Assessment Model (GCAM). For instance, AIM simulates future regional land use dynamics by categorizing the world into 17 geopolitical regions^4^, as compared to a classification of 26 world regions in the MAGNET model^5^. Therefore, IAM-based LULC projections suffer from a lack of geospatial details or a coarse resolution^5–7^. To improve the spatial resolution, the Land Use Harmonization project as part of the CMIP6 produced future-scenario-based global LULC on a 0.5° grid for Land Use Harmonization Version 1 (LUH1)^8^ and later boosted the spatial resolution to 0.25° for LUH2^9^. Chen *et al*.^10^ further refined the spatial information of global LULC from 2015 to 2100 under an enriched package of SSP-RCP scenarios by developing the Demeter tool, and downscaled future land use demands obtained from GCAM to a 0.05° resolution. Yet, the spatial resolution in these studies, ranging in a wide spectrum from five arc minutes to 0.5° ^8–14^, is not sufficient to capture the geographic heterogeneity of land use dynamics. As clarified by Li *et al*.^15^, some widely-adopted land cover products even at a grid scale of 10 km may yield substantial deviations when depicting the geospatial changes of land cover patterns, which may in particular cause severe distortions to global urban land patterns^16^. Moreover, the deviations in LULC projections may cause a spread of uncertainty in earth system models (ESMs), thus jeopardizing the accuracy of ESM output data as well as the applicability of land use products^11,17^.

Cellular automata (CA) have proved to be efficient in allocating land use to more spatially-explicit details, which are capable of defining the rules of cell-to-cell transformation as well as their adaptive behaviors and thus simulating the complex geospatial patterns. By integrating with land use demands generated from IAMs, CA models are capable of simulating scenario-based gridded LULC datasets with a finer resolution (normally 1 km). For instance, Li *et al*.^15^ generated a future land use dataset (2010–2100) by applying the FLUS model to downscale the land-use demands of 17 global regions in the IMAGE model on a 1 km grid. A recent effort by Chen *et al*.^18^ has moved a further step to integrate land use demand data from LUH2 into 31 geopolitical regions and then used the FLUS model to downscale the regional land-use data to a 1 km resolution under typical SSP-RCP scenarios. Yet, recent works^15,19^ highlighted that using the land use demand of highly-integrated geopolitical regions generated from IAMs (such as the 17-region classification in IMAGE and 31-region classification adopted in the abovementioned study) for spatial downscaling may fail to preserve the geographically heterogeneous characteristics of global land patterns, given that land use patterns may differ substantially across subregions that are geopolitically integrated but with totally different climate and soil conditions.

Moreover, existing practices of future land use allocation generally utilize historical baseline to simulate land use dynamics under representative SSP-RCP scenarios, overlooking the effects of the simulated results of the former year on subsequent ones^18,19^. Additionally, previous studies generally rely on the empirical relationship between historical driving factors and land use, neglecting that the correlation is subject to variations in climatic and socio-economic factors. According to the recent work by Chen *et al*.^20^, existing research utilizing CA models for LULC simulation often lacks the ability to reflect the potential future variations in the relationship between driving forces and land use owing to climate change. In the foreseeable future with intensified climate change, the climate zones encompassing almost half of the world’s land surfaces may undergo drastic changes^21,22^. Hence, the accuracy of future land use dynamics, especially those natural land classes like vegetations that are particularly susceptible to climate change effects^20^, may be largely jeopardized if we persist on adopting historical suitability probability for allocating future land use demand to a higher spatial resolution.

This study aims to produce a gridded dataset of global LULC at 1 km × 1 km resolution under typical SSP-RCP scenarios from 2020 to 2100 by combining the Patch-generating Land Use Simulation (PLUS) model and GCAM. Firstly, we use GCAM to simulate global land use demand at the water-basin level and calibrate urban land use generated from GCAM by means of a multivariate regression method. Compared to other IAM models such as IMAGE and AIM, GCAM has its advantages in simulating the spatial heterogeneity of global LULC with a classification of 235 regions at the water-basin level, by taking into account the discrepant land cover patterns in different agro-ecological zones and diversified socio-economic levels of different geopolitical regions. Secondly, by using the PLUS model to simulate the patch-level changes of various land classes driven by future suitability probabilities, we allocate the regional land use demand in various future scenarios to a spatial resolution of 1 km × 1 km. Finally, we compare the gridded dataset for future LULC with existing land use products to validate the accuracy of the results. This new dataset guarantees consistency across different SSP-RCP scenarios at explicit spatial details. Moreover, compared to other LULC products for future periods, this gridded dataset fully incorporates the impact of simulated results on temporally adjacent simulations and enhances the fidelity by using future suitability probabilities for land use projection. The outcome of this study can greatly help improve the accuracy of simulating earth system dynamics and modeling human-earth interaction.

## Methods

### Overall framework

**Fig. 1:**
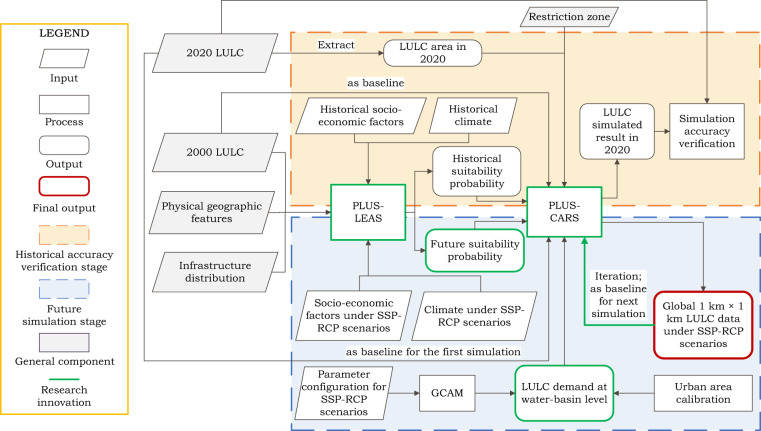
Overall framework of LULC simulation under various SSP-RCP scenarios.

Figure 1 shows the overall framework for simulating future global LULC. First of all, the global development pathway (SSP-RCP) parameters in the context of climate change were input into the GCAM model to predict the total area (called “demand”) of each LULC type at the water-basin scale under future scenarios. This was followed by allocating land use demands within each basin to spatially-explicit details using the PLUS-LEAS model. Regarding accuracy verification for historical periods, both historical land use data and associated driving factor data (see Table 1), which have been resampled to 1 km resolution and transformed into the Mercator projection, were fed into the PLUS-LEAS model to generate the historical suitability probability. By taking the quantity of actual land use as demands and giving full account to the competition between different land classes as well as LULC growth constraints under the demand-driven principle, we adopted PLUS-CARS as a CA-based model to downscale the historical land use demand on a 1 km × 1 km grid. The simulated results were then cross-verified for accuracy with actual data. Regarding accuracy verification for future periods, future suitability probability maps were generated using historical and future driving factors. These are incorporated into the PLUS-CARS model in conjunction with land use data from the preceding phase of the simulation period, predicted future LULC demand, and restriction zone data, which allows for the spatial-temporal dynamic simulation of future global land use/cover pattern changes.

**Table 1:** Data sources and descriptions.

Category	Data	Year	Resolution/Scale	Source
Land	Land use/Land cover	2000, 2020	300 m	ESA-CCI^30^
Socio-economic data	Population density	2020	1 km	GPW v4.11^59^
	Gross domestic product	2019	1 km	Chen *et al*.^60^
	Future population under SSPs	2020–2100	0.5°	Jiang *et al*.^35,36^
	Future gross domestic product under SSPs	2020–2100	10 km	Murakami *et al*.^39,40^
	Roads, railways and cities		vector (1:10million)	Natural Earth (http://www.naturalearthdata.com)
Climate change	Annual precipitation	2019	0.25°	NOAA GPCC^61^
	Annual mean temperature	2020	0.5°	NCAS CRU TS^62^ (https://crudata.uea.ac.uk/cru/data/hrg/cru_ts_4.06/cruts.2205201912.v4.06/tmp)
	Annual precipitation and mean temperature under SSP-RCPs	2020–2100	0.25°	NEX-GDDP-CMIP6 (https://registry.opendata.aws/nex-gddp-cmip6)
Physical data	Soil type	2013	vector	HWSD (https://www.fao.org/soils-portal/soil-survey/soil-maps-and-databases/harmonized-world-soil-database-v12)
	Lakes and rivers		vector (1:1million)	CAS (https://www.resdc.cn/)
	DEM	2008	1 km	NOAA GLOBE Topography (https://www.ngdc.noaa.gov/mgg/topo/DATATILES/elev)
Protected areas	Protected area boundary	2022	vector	WDPA (https://www.protectedplanet.net)

### Projection of LULC demand

GCAM is an open-source, multi-sector coordinated IAM developed by the Pacific Northwest National Laboratory (PNNL), which gains wide applications in modeling agriculture and land use^10,19^, water supply and demand^23^, and greenhouse gas emissions^24,25^. GCAM harmonizes and simulates the behavior and interaction between five systems: the energy system, water, agriculture and land use, economy and climate. Initially, GCAM reads external scenario assumptions of key drivers (such as population, economic activity, technology and policies) and then assesses the impact of these assumptions on key decision-making-related outcomes (e.g., commodity prices, energy use, land use, water use, and emission concentrations)^26,27^. GCAM operates within a time frame from 1990 to 2100 with 5-year time intervals^27,28^. Spatially, GCAM divides the world into 32 geopolitical regions and 235 water basins, as seen in Figure S1. Land use simulation on the water-basin level can fully reflect the interactions between the earth system and anthropogenic activities. Compared with the grouping system of previous LULC simulations (a classification of 32 integrated geopolitical regions^18^ or a 17-region classification^15^), dividing the world into 235 water basins provides a refined representation of how spatial heterogeneity at the macro scale affects the LULC simulation.

We put the global development path assumptions of typical SSP-RCP scenarios (SSP1-2.6, SSP2-4.5, SSP3-7.0, SSP4-3.4, SSP5-8.5) into the GCAM model to obtain future quantity demand for various types of land use/cover in 235 water basins. In terms of SSP-RCP scenarios, SSP1 indicates a path of sustainable development; SSP2 represents an intermediate development trajectory close to historical trends; SSP3 reflects a rugged path influenced by regional competition; SSP4 characterizes a development path of pronounced inequality and stratification; and SSP5 signifies a rapid development path steered by intensive fossil fuel consumption. The corresponding RCPs depict the representative concentration paths that may occur in each SSP scenario. Moreover, since the urban area in the GCAM model remains static, we use future GDP and population output produced by the GCAM model under various SSP-RCP scenarios to calibrate the projected demand of future urban areas, based on the multiple regression method put forward by Dong *et al*.^29^. We take the historical (2000 and 2020) ESA-CCI LULC data^30^ as the simulation baseline and establish a mapping relationship between ESA-CCI data^30^ and the corresponding demand output from GCAM (refer to Table S1). As a result, we are able to match and calibrate the predicted LULC demand by GCAM with ESA-CCI data^30^ in terms of both land use types and quantity.

### Downscaling of LULC demand on a water-basin scale

Spatially explicit LULC simulation models provide a means to convey changes from macro to local scales^29^. We used the CA model PLUS^31^ to downscale the calibrated LULC quantity demand, validate the accuracy of the projections, and simulate the spatially-explicit distribution of future LULC. The PLUS model, introduced by Liang *et al*.^31^ in 2021, is a CA model that simulates LULC changes with a patch-generating simulation strategy. Compared to CA models like CLUE-S, CA-Markov, and FLUS, the PLUS model demonstrates improvement in the following aspects. Firstly, it incorporates the novel Land Expansion Analysis Strategy (LEAS). The transformation rules obtained from LEAS have temporal characteristics that can describe the land cover change characteristics during specific periods, facilitating a more comprehensive exploration of the driving factors behind changes in various LULC types. On this basis, PLUS employs the Random Forest algorithm to establish relationships between various land use expansions and their driving forces. This not only enables the acquisition of suitability probabilities for each LULC type but also facilitates a quantitative exploration of the roles that driving factors play in the expansion of LULC. Secondly, the PLUS model includes a CA model based on multi-type Random patch Seeds (CARS). CARS represents an enhancement of traditional CA models within the PLUS framework, incorporating a patch generation mechanism that utilizes multiple types of random seeds based on threshold decline. This mechanism exhibits spatial-temporal dynamics that enable the spontaneous and unrestricted growth of new LULC patches under the constraints of combined suitability probabilities. CARS has its strength in simulating the change in multi-class LULC at the patch level, including forests, grasslands and other natural LULC types. With the aforementioned improvements, PLUS can autonomously generate land patches within spatiotemporal dynamics, thereby ensuring consistency with the principles of landscape evolution^31^. In our paper, the downscaling process conducted using the PLUS model can be divided into historical accuracy verification and future simulation stages.

1. Accuracy validation for historical periods: For accuracy validation, we firstly employed the LEAS of the PLUS model to extract the expansion grids of each LULC type from historical LULC data for the years 2000 and 2020. Then we used the random forest model to generate the historical suitability probability of each LULC type and analyzed the contribution rate of various driving factors to the expansion of specific land cover types. Subsequently, based on the land cover demands obtained from historical ESA–CCI LULC data^30^ and the generated historical suitability probability, the CARS module of the PLUS model was employed to simulate LULC changes in 2020 using historical LULC data from 2000 as the baseline. The competition of LULC types is influenced by adaptive coefficients, promoting the LULC quantity to meet the future demand in the simulation process. To achieve the optimal performance of the PLUS model, we employed the method of controlling variables to set parameters related to random forest modeling and patch morphological regulation (see Text S1 for details). In addition, we incorporated spatial distribution data of water bodies (lakes and rivers) and protected areas as restriction zones for LULC growth.
  Upon obtaining the simulated LULC distribution for 2020, we calculated the Overall Accuracy (OA), Kappa coefficient, and Figure of Merit (FoM) of LULC simulation in each water basin to complete the accuracy validation and proceed to the simulation stage for future periods. Whereas OA is the ratio of correctly classified land cells to the total number of cells; the Kappa coefficient is a metric derived from OA and is used to further assess the consistency between the simulation result of the downscaling model and the actual land use pattern; FoM signifies the proportion of accurately predicted LULC changes relative to the combined sum of observed and predicted changes, which is generally utilized as a supplement to Kappa coefficient in evaluating the accuracy of LULC change simulations^32–34^ (see Text S2 for details).
2. Simulation for future periods: For each water basin, we first replaced the historical data of temperature, precipitation, population density and GDP with corresponding data for future periods. The updated data were then inputted into the random forest model to generate the future suitability probability of each LULC type from 2030 to 2100. Using the historical LULC data in 2020 as the simulation baseline, we simulated the spatially-explicit LULC in 2030 based on the generated probability map and the quantity demand for each LULC type, by taking into account restriction zone data. Subsequently, using the simulated LULC in 2030 as the baseline, we iteratively employed the generated future suitability probability and LULC quantity demand for the subsequent simulation period, while adhering to the restriction zone limitations. This iterative process continued until the completion of the LULC simulation for 2100.

The baseline LULC data, driving factor data, and restriction zone data used in the simulations are described as shown in Table 1. Specifically, the future gridded population datasets under SSPs come from Jiang *et al*.^35^, which are generated using the Population-Development-Environment (PDE), a methodology that gives full consideration to fertility, mortality, and migration by educational levels, while accounting for age and gender-related transitions^36–38^. The data representing global gridded GDP distributions^39^ in accordance with the five SSPs are generated by Murakami *et al*.^38^, through a methodology that integrates spatial econometrics, urban growth patterns modeling, and auxiliary geographic data^40^. The global temperature and precipitation data under future SSP-RCP scenarios were derived from the bias-corrected NASA global daily downscaling prediction (NEX-GDDP-CMIP6) dataset (https://registry.opendata.aws/nex-gddp-cmip6) with a daily temporal resolution and a spatial resolution of 0.25° (approximately 25 km)^41^. In accordance with the criteria of a comprehensive coverage of climatic elements and a fine spatial resolution (<100 km), we selected 11 global climate models (GCMs) from 35 GCMs covered by the dataset, including BCC-CSM2-MR, CMCC-ESM2, EC-Earth3, EC-Earth3-Veg-LR, GFDL-ESM4, INM-CM4-8, INM-CM5-0, MPI-ESM1-2-HR, MRI-ESM2-0, NorESM2-MM, TaiESM1. By averaging the models and daily data, we obtained the multi-model ensemble average data for future annual mean temperature and total precipitation.

## Data Records

The dataset generated in this study is on a global scale with a resolution of 1 km and encompasses a timespan from 2020 to 2100, which is publicly available on 10.6084/m9.figshare.23542860^42^. These data are projected in the world-Mercator projection coordinate system and are provided in single-band GeoTIFF format, which can be easily utilized by various mainstream GIS and RS platforms such as ArcGIS, QGIS, ENVI, as well as programming languages such as Python and MATLAB. The simulated data files follow a standardized naming convention “sspx_pp_yyyy.tif”, where x represents the simulated SSP scenario (1 to 5), pp represents the simulated RCP scenario; and yyyy represents the simulated year. For example, the data file named “ssp1_26_2030.tif” corresponds to the LULC simulation data for the year 2030 under the SSP1-2.6 scenario. Each GeoTIFF data file includes integer raster attribute values ranging from 1 to 6, which represent the following land use types: cropland, forest, grassland, urban, barren, and water. In addition, the dataset also covers the simulated LULC in 2020 according to the overall simulation framework of this study. Figure S2 illustrates the spatial distribution of simulated LULC data by taking 2030, 2050, 2070 and 2100 as reference years.

## Technical Validation

### Accuracy for land use simulation

**Fig. 2:**
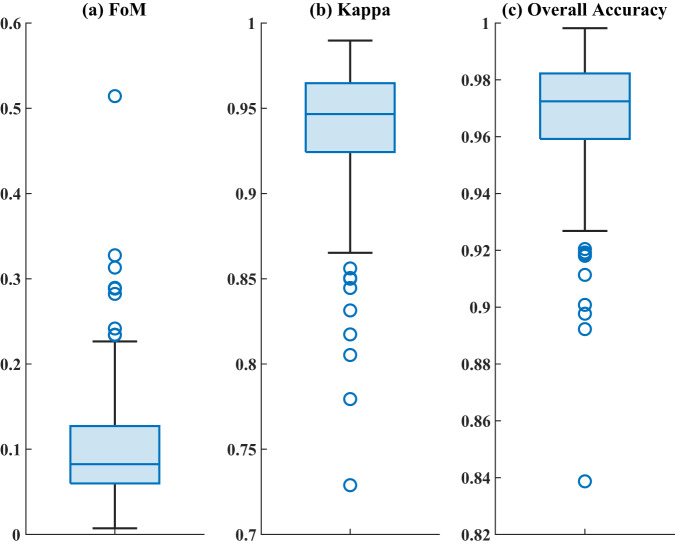
Box diagram of FoM, Kappa coefficient and OA in global water-basin-level regions.

The accuracy validation for historical simulation results shows that the average FoM, Kappa coefficient and OA across different water basins are 0.10, 0.94, and 0.97 (see Table S2), respectively. Compared with the simulation accuracy (FoM = 0.10, Kappa coefficient = 0.87, OA = 0.93) of the 1 km × 1 km LULC dataset under SSP-RCP scenarios at a global scale—*Global LULC dataset*^18,43^, this study demonstrates a comparable FoM value, but a higher value of Kappa coefficient and OA. From the spatial distribution of FoM values in global water basins simulated in this study (see Figure 2, Figure S3, and Table S2), the FoM of each water basin is within the normal range (1–59%)^33^. To further illustrate our findings, we use China as a specific case study. In this work, the FoM values in China (refer to Figure S3 and Table S2) range between 0.06 and 0.22, with an average of 0.12. This aligns closely with the accuracy of an existing LULC simulation of China (FoM ∈ [0.10, 0.17], $\overline{FOM}$ = 0.13)^18,44^. Besides, the optimal ratio between FoM and the percentage of changed LULC pixels is ideally above 1.5:1^33^. In this study, the percentage of changed LULC pixels during 2000–2020 is 2.2%, with the ratio of FoM to the percentage of pixel change reaching 4.5:1. These validation results clearly demonstrate that our dataset’s simulation accuracy for LULC is within an acceptable range, which underscores the reliability of our approach.

We also calculated Pearson correlation coefficients to further assess the spatial conformity between the simulated results and historical LULC data in 2020, considering both overall and subtype-specific LULC distributions (refer to Table 2). The correlation coefficient for the overall LULC across the two datasets reaches 0.97 when not distinguishing LULC types. When focusing on natural LULC categories such as cropland, forest, and grassland, the correlation coefficients range between 0.89 and 0.97. This indicates that our simulation results align well with the historical data for both overall LULC and natural LULC types.

**Table 2:** Correlation coefficients between the simulation results of the dataset produced in this study and the actual historical data from ESA-CCI^30^ in 2020.

LULC type	Pearson’s correlation coefficient
All	0.969
Cropland	0.961
Forest	0.946
Grassland	0.889
Urban	0.685
Barren	0.969
Water	0.974

**Fig. 3:**
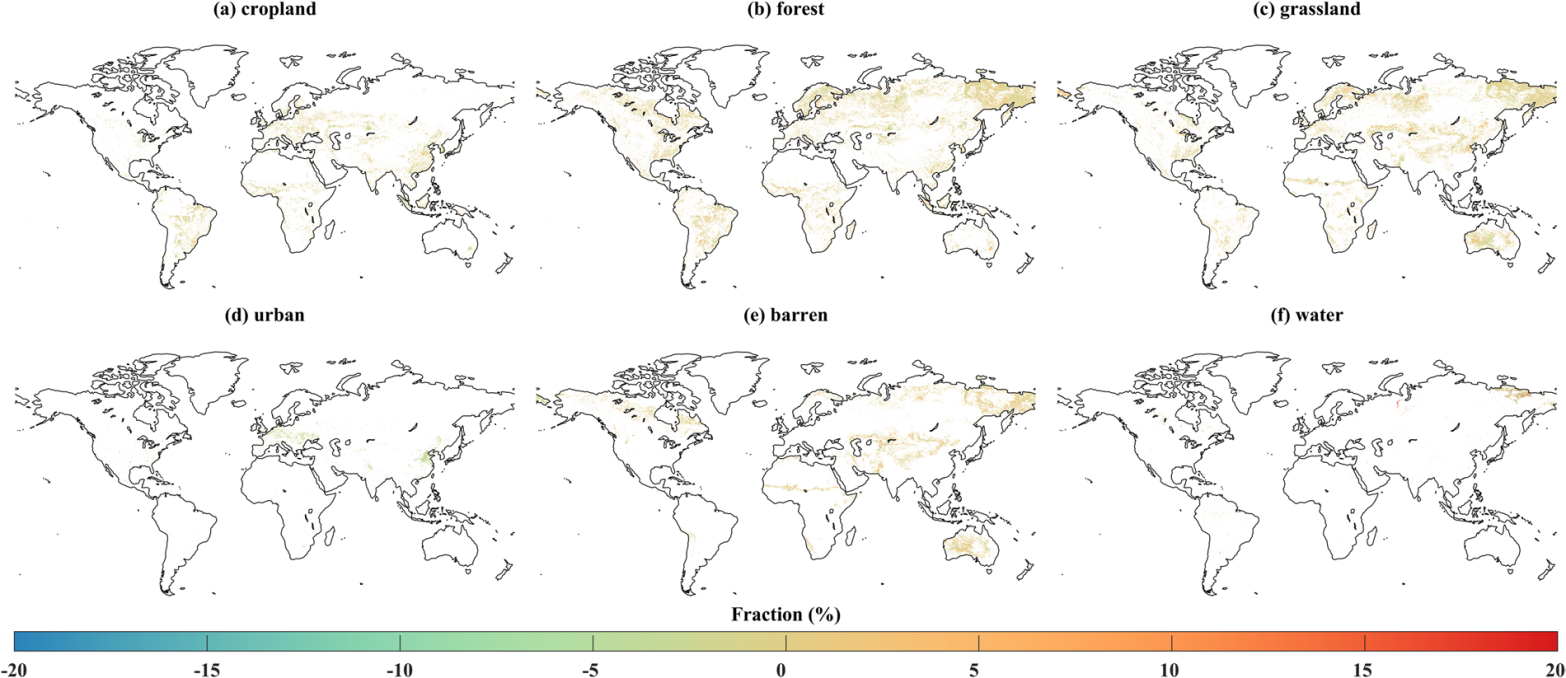
Area difference between the simulated LULC and historical data for the year 2020. Values indicate the proportional change in LULC within each 10 km × 10 km grid.

Moreover, Figure 3 illustrates the gap in the area within each 10 km × 10 km grid between the simulation results of this study and the historical LULC in 2020. The discrepancy for each LULC type ranges from 0 to 20%, which is within the normal range when compared with the existing research (0–50%)^45^. To sum up, the LULC simulation framework in this study demonstrates its capacity to accurately capture the global spatial distribution of LULC.

### Suitability probability generated by driving factors for future periods

**Fig. 4:**
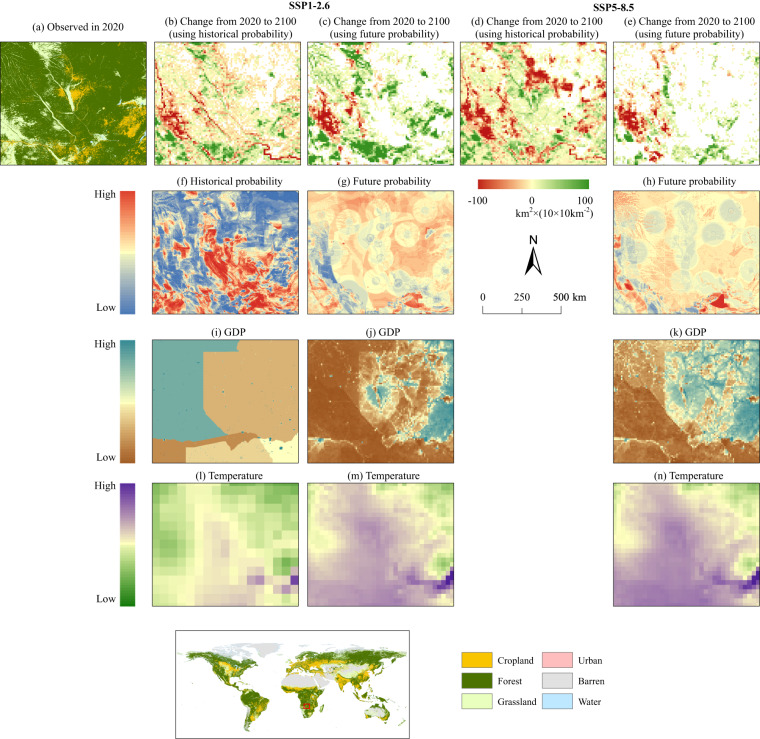
Forest expansion simulation using historical and future suitability probabilities. (**a**) shows the distribution of observed LULC in the case region in 2020; (**b**–**e**) show the change in forest area from 2020 to 2100; (**f**–**h**) show the historical and future suitability probability for forests under historical and future scenarios; (**i**–**n**) show the spatial distribution of driving factors under historical and future scenarios.

In the simulation stage for future periods, we found that the LULC simulation derived from future suitability probability can effectively capture the influence of driving factors on LULC change. Using the forest in Southern Africa as a case, Figure 4 compares the historical suitability probability (see Figure 4f) to the future suitability probability (see Figure 4g,h), and illustrates the forest area change from 2020 to 2100, which were generated using both the historical suitability probability (see Figure 4b,d) and the future suitability probability (see Figure 4c,e). According to the basin-specific maps of dominant driving factors for LULC changes that are produced using the random forest model (see Text S3 and Figure S8), we found that GDP and temperature emerge as the principal socio-economic and climate factors driving the forest expansion in southern Africa. Figure 4 incorporates the historical (see Figure 4i,l) and projected future (see Figure 4j,k,m,n) distributions of the two drivers, which shows how these factors shape the generation of future suitability probability.

Our analysis revealed that the future suitability probability has evolved from its historical counterpart due to the influence of future driving factors. Regions with lower suitability probabilities in the historical probability landscape (shown in blue in Figure 4f) are expected to see a general increase in the future period (see Figure 4g,h). As a result, most forest areas that are simulated to decrease using the historical suitability probability (see Figure 4b,d) are predicted to increase under the influence of future suitability probability (see Figure 4c,e). Different from the apparent forest expansion in the SSP1-2.6 scenario (see Figure 4c), the majority of the forest area under the SSP5-8.5 scenario is expected to remain unchanged (blank in Figure 4e). This can be ascribed to the overall lower suitability probability under the SSP5-8.5 scenario (see Figure 4h) as compared to that under the SSP1-2.6 scenario (see Figure 4g).

We further examined the distribution of the driving factors that lead to the difference between historical and future suitability probability. Historically, the GDP level displayed noticeable national differences. Given Angola’s higher historical economic level in comparison to Zambia (see Figure 4i), the traditional discord between economic benefits and forestry protection^46^ has resulted in a diminished suitability probability for forest expansion relative to Zambia^47^. However, in the SSP1-2.6 scenario (see Figure 4j), the economic level of eastern Zambia is projected to advance by 2100, which precipitates a decrease in forest suitability probability in this region (see Figure 4g). In contrast, the lower GDP level of western Zambia yields a higher suitability probability than its eastern counterpart. In the SSP5-8.5 scenario, Zambian is projected to see further growth in GDP level (see Figure 4k). In that case, the forest suitability probability will dwindle compared with that in the SSP1-2.6 scenario (see Figure 4h).

However, the low forest suitability probability observed in the northeastern part of the case region (within Zambia, as depicted in Figure 4f) indicates that the socio-economic factor can only partially explain the suitability probability. The effect of climatic factors, such as temperature, on forest suitability probability should also be taken into account. Existing research underscores a positive correlation between forest growth and the rise in temperature/carbon dioxide fertilization in Africa^48,49^. This indicates that the historically low forest suitability probability in the northeastern part of the case region (see Figure 4f) can be partly attributed to the relatively low temperature (see Figure 4l). By the end of the 21st century, the temperature within the case region (see Figure 4m,n) is predicted to exceed historical levels (see Figure 4l), resulting in a general increase in the forest suitability probability (see Figure 4g,h) compared with its historical counterpart (see Figure 4f). Nonetheless, the future suitability probability in the middle of the case region is projected to decline, which is mainly attributed to the economic enhancement (GDP) in the future (see Figure 4j,k). This demonstrates that the future climate and socio-economic factors have an overall and substantial impact on LULC change. Therefore, in LULC simulations, it is pivotal to take into account the change in suitability probability based on the evolution of driving factors. Relying solely on the spatial distribution of historical driving factors to generate suitability probability will inevitably overlook the impacts of future climatic and socio-economic changes on LULC.

### Variations in global LULC changes across different SSP-RCP scenarios

**Fig. 5:**
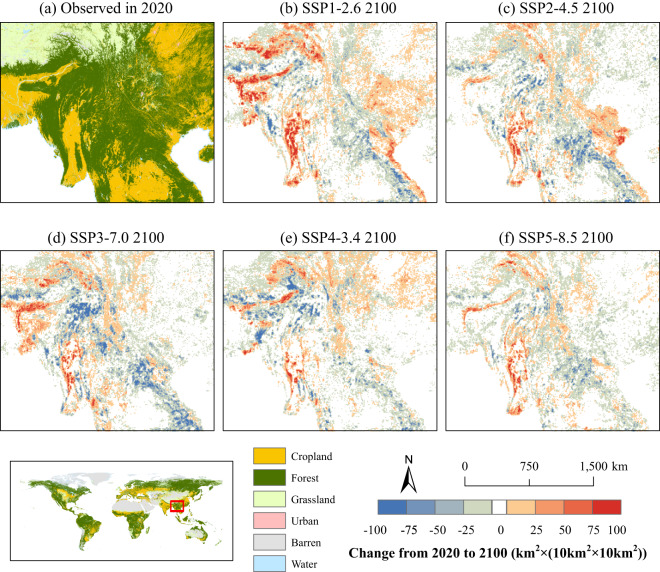
The spatial distribution of changes in forest area from 2020 to 2100 in Southeast Asia under future scenarios. (**a**) shows the spatial distribution of LULC in Southeast Asia in 2020; (**b**–**f**) show the forest area changes from 2020 to 2100 under each SSP-RCP scenario.

The discrepancy in LULC under various SSP-RCP scenarios is well reflected in the dataset produced in this study. By taking Southeast Asia as an example, Figure 5 shows changes in forest area across various SSP-RCP scenarios during 2020–2100 in Southeast Asia (where red indicates forest increase, and blue corresponds to forest decrease). In the SSP1-2.6 scenario (see Figure 5b), the sustainable development pathway will have an obvious effect on Southeast Asia, thereby mitigating deforestation and facilitating forest protection and expansion. This scenario represents the most evident forest expansion, primarily attributed to improvements in agricultural efficiency and policies encouraging the transformation of farmland to forest^50^. In contrast, in the SSP3-7.0 scenario (see Figure 5d), Southeast Asia experiences the most pronounced forest area loss transiting to cropland (see Figure S15), when compared to other scenarios. This transition is predominantly affected by regional competition, national land tenure policies to ensure domestic food supply^51^, and the lack of environmental protection awareness^50^. When compared with the SSP3-7.0 scenario, the decline in forest area within Southeast Asia under the SSP2-4.5 scenario (see Figure 5c) and SSP4-3.4 scenario (see Figure 5e) is mitigated by afforestation initiatives targeting emission reduction and investments towards a low-carbon economy, respectively^50,52^.

**Fig. 6:**
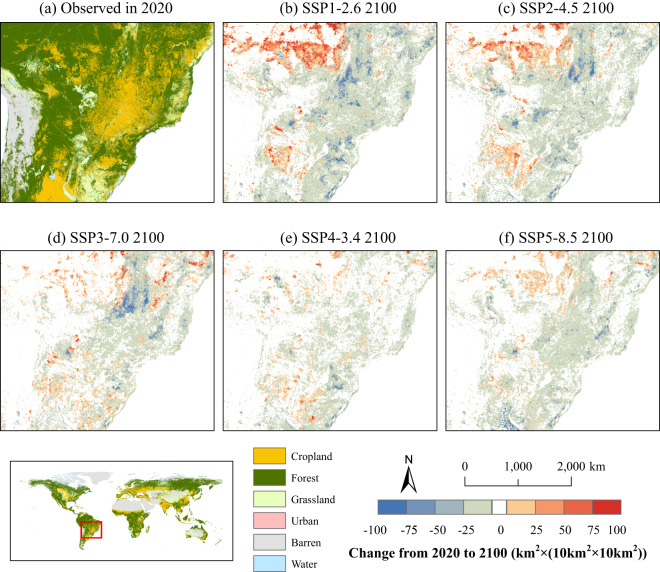
The spatial distribution of changes in cropland area from 2020 to 2100 in South America under future scenarios. (**a**) shows the spatial distribution of LULC in South America in 2020; (**b**–**f**) show the cropland area changes from 2020 to 2100 under each SSP-RCP scenario.

We take the grain-producing region of Brazil in South America as a case region to analyze the cropland change under various future scenarios (refer to Figure 6). Under the SSP1-2.6 (see Figure 6b) and SSP2-4.5 (see Figure 6c) scenarios, an evident expansion of cropland is observed in northwestern Brazil and southern Amazon as well as the Cerrado, primarily accompanied by an invasion of forest and grassland areas. This region, known as the “arc of deforestation”, is the transition zone between forest and cropland. Under these two scenarios, the rapid global economic development, coupled with intensive demands for food imports (such as soybeans) from other countries, boosts the deforestation trend^52–55^. At the same time, the sustainable development path will increase the demand for biomass energy crops such as sugar cane^56^. Thus, the expansion of cropland within the arc of deforestation is largely driven by economic development and national policies (see Figure S9). Nevertheless, Brazil’s grain-producing areas tend to shrink slightly in future scenarios, especially in the SSP3-7.0 scenario (see Figure 6d). This may be attributed to hampered international trades that weaken demand for food exports. Moreover, national goals aiming to ensure national food security may further stimulate agricultural intensification^56^.

**Fig. 7:**
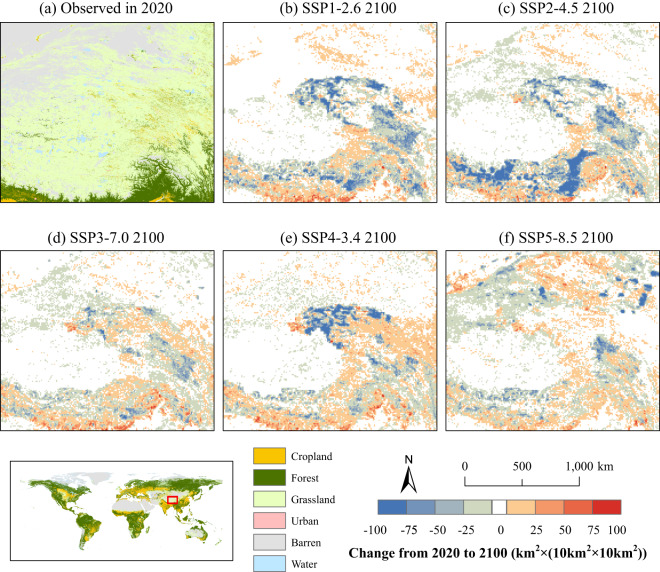
The spatial distribution of changes in grassland area from 2020 to 2100 in the Tibetan Plateau in future scenarios. (**a**) shows the spatial distribution of LULC in the Tibetan Plateau in 2020; (**b**–**f**) show the grassland area changes from 2020 to 2100 under each SSP-RCP scenario.

To illustrate the variations in grassland areas across different future scenarios, we concentrate our focus on the Tibetan Plateau, a representative steppe region (see Figure 7). Under the SSP1-2.6 (see Figure 7b), SSP2-4.5 (see Figure 7c), and SSP4-3.4 (see Figure 7e) scenarios, a substantial decline in grassland areas is witnessed. In the sustainable development pathway as depicted in the SSP1-2.6 scenario, reduced consumption of animal products and lower demand for pastures precipitate a considerable transformation of grasslands into forests (refer to Figure S17b). These forest expansions are mostly prominent in the eastern Tibetan Plateau, where the hydrothermal conditions are particularly conducive to such transformations. The SSP2-4.5 scenario forecasts an increase in suitable cultivation areas for wheat and other food crops on the Tibetan Plateau^57^, particularly within the Yarlung Zangbo river basin (refer to Figure S17c). Under the SSP5-8.5 scenario (see Figure 7f), severe climate warming on the Tibetan Plateau may result in a rise in the desertification area in its northwest region^58^. Along with this environmental shift, intensive animal husbandry is forecasted to persist in this region, which is characterized by a growing population with an unabated demand for animal products and feed. This interplay of socio-economic and environmental factors contributes to relative stability in grassland area extent, forestalling any drastic reductions.

### Comparison with other LULC datasets

We compared the gridded dataset generated in this study to LUH2^9^ (openly available at https://luh.umd.edu/data.shtml) and *Global LULC dataset*^18,43^, which serve two representative LULC datasets that were produced across different SSP-RCP scenarios (for the classification and mapping relationships among these datasets, refer to Table S1). We first analyzed the correlation between our dataset and the *Global LULC dataset*^18,43^ at representative temporal junctures under future scenarios, with the correlation coefficients shown in Table 3. The results show that the correlation coefficients of the two datasets range from 0.87 to 0.89 for each scenario and typical year, which demonstrates the capability of the dataset of this study in simulating the future spatial distribution of LULC.

**Table 3:** Global average Pearson correlation coefficients between the dataset of this study and the *Global LULC dataset*^18,43^ at typical temporal junctures under future scenarios.

		Pearson’s correlation coefficient			
		2030	2050	2070	2100
**SSP-RCP Scenarios**	**SSP1-2.6**	0.887	0.882	0.879	0.876
	**SSP2-4.5**	0.887	0.884	0.881	0.878
	**SSP3-7.0**	0.886	0.883	0.880	0.877
	**SSP4-3.4**	0.886	0.881	0.876	0.869
	**SSP5-8.5**	0.887	0.884	0.881	0.878

**Fig. 8:**
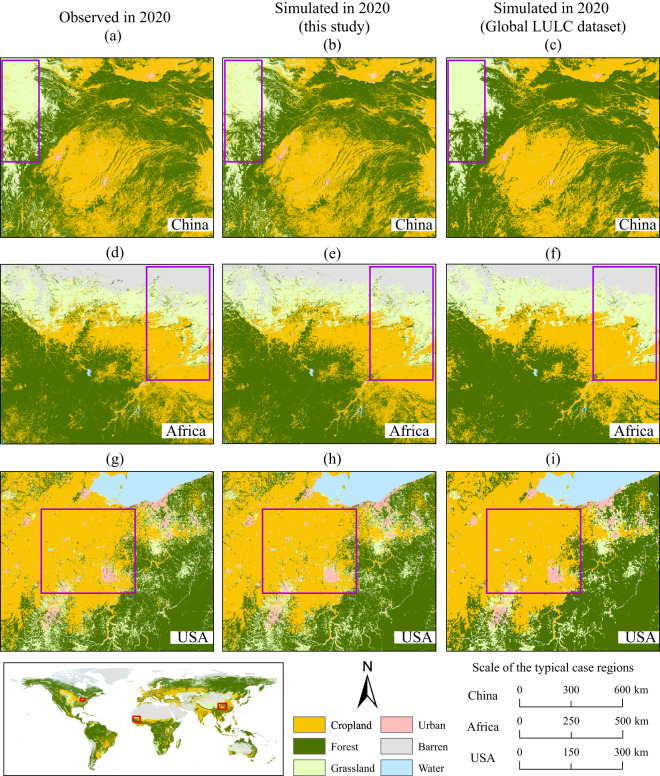
Comparison of LULC for the year 2020 in typical case regions of China, Africa and the United States between ESA-CCI data^30^, the dataset produced in this study, and the *Global LULC dataset*^18,43^. Since the year 2020 is taken as the future simulation period in the *Global LULC dataset*^18,43^ and is modeled under different SSP-RCP scenarios, here we take the simulated LULC result for 2020 under the SSP2-4.5 scenario from the *Global LULC dataset*^18,43^ for comparison, because this scenario follows the historical development path and is close to the actual historical development.

Selecting regions with comprehensive LULC types in China (FoM = 0.12), Africa (FoM = 0.08), and the United States (FoM = 0.11) as examples, we validated the simulation accuracy by comparing our simulation results (see Figure 8b,e,h), the *Global LULC dataset*^18,43^ (see Figure 8c,f,i), and the observed LULC data from ESA-CCI^30^ (see Figure 8a,d,g) in 2020. Figure 8 shows a high degree of consistency between the distribution pattern and simulation precision of our dataset and the historical data. This is because the CA model used in this study (PLUS-CARS) can accurately simulate the patch growth of natural LULC types on a fine scale. However, the CA model used in preparing the *Global LULC dataset*^18,43^ (FLUS) may be comparatively less robust in the patch-level simulation, leading to the disappearance of small patches of some LULC types (as evidenced in the purple boxes of Figure 8) and in turn reducing the accuracy of simulating intricate details.

**Fig. 9:**
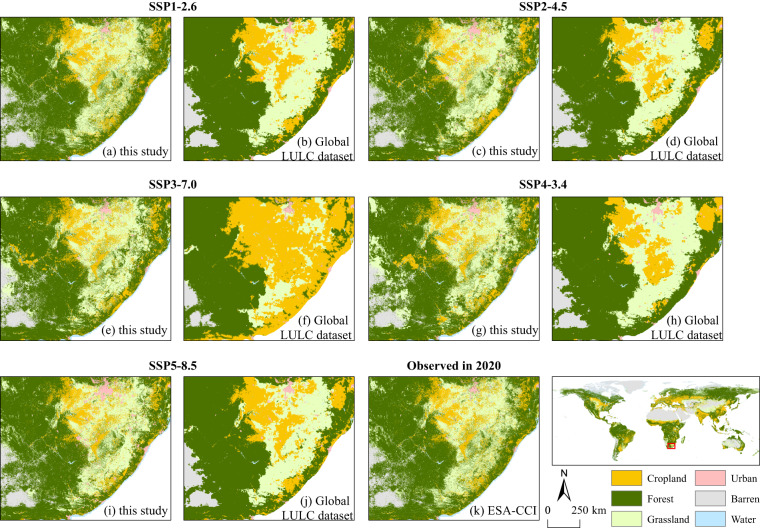
Comparison of the simulated LULC result of a typical case region (within South Africa) in 2100 under different SSP-RCP scenarios between the dataset produced in this study and the *Global LULC dataset*^18,43^.

Figure 9 presents a comparison between the dataset of this study and the projection of the *Global LULC dataset*^18,43^ for the year 2100 under various future scenarios. The results underscore the capacity of our dataset to provide explicit spatial detail in simulating LULC distributions across a range of future scenarios in 2100. This could be attributed to two aspects. First, in contrast to the *Global LULC dataset*^18,43^, which divided the globe into 32 regions for the downscaling process, we divided the world into 235 regions at the water-basin level. This approach provides a better representation of the spatial heterogeneity in LULC simulations, and facilitates more precise control over the allocation of LULC demand within each water basin. Secondly, we adopted an iterative simulation technique starting with the historical data from 2020 as the basis for modeling the LULC of the subsequent future period, which was then used as the foundation for the simulation of the next period. This iterative approach considers the spatial impact of previous LULC results on the simulation of subsequent periods. By limiting the spatial and temporal scope of the simulation, we were able to control the number of pixels undergoing changes within each water basin. These abovementioned strategies help prevent the uncontrollable expansion of changing pixels within larger regions over extended simulation periods, and thus avoid the aggregation of changing pixels in areas with high suitability probability (refer to Figure 9 and Figure S18).

**Fig. 10:**
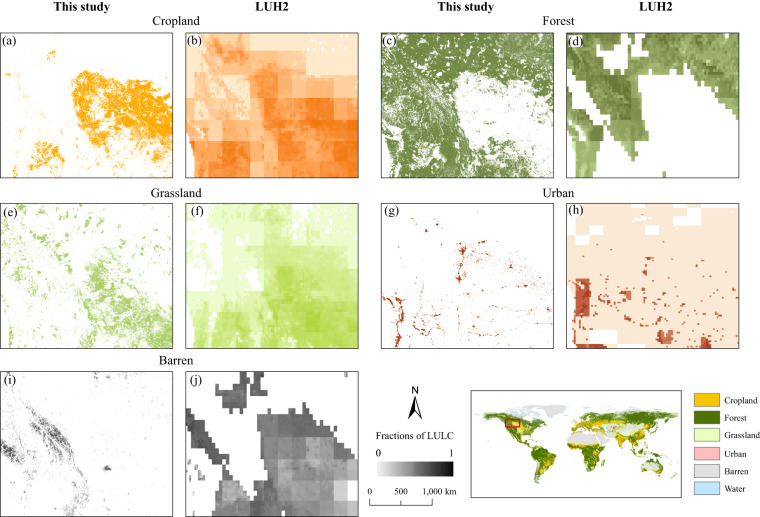
Comparison of the simulated LULC result of a typical case region (within North America) in 2100 under the SSP4-3.4 scenario between the dataset produced in this study and LUH2^9^.

Figure 10 compared the LULC map produced in this study to the gridded fraction map from LUH2^9^ for the year 2100 under the SSP4-3.4 scenario. We selected this scenario as both datasets, ours and LUH2^9^, are underpinned by land use demand data derived from GCAM^52^, thereby ensuring the consistency of IAM. It can be seen from Figure 10 that a high degree of spatial consistency between the two datasets across all LULC types is revealed for the case area of North America, indicating that the dataset produced in this study effectively captures the spatial distribution of LULC provided by LUH2^9^. Moreover, the LULC dataset produced in this study demonstrates advantages in terms of geospatial details, which has a spatial resolution of 1 km as compared to the 0.25° resolution in the LUH2 dataset^9^. This allows for a finer and more accurate representation of the LULC landscape under various future scenarios.

## Usage Notes

This study produced a global-scale LULC dataset that includes 6 LULC types with a 1 km resolution from 2020 to 2100 under different SSP-RCP scenarios. The dataset displays robust simulation accuracy across world regions at the water-basin level (FoM = 0.10, Kappa coefficient = 0.94, OA = 0.97), which can efficiently capture the differential impacts exerted by each SSP-RCP scenario on the future expansion of LULC. This LULC dataset is fully supportive for mapping the geospatial heterogeneity of future land use and can provide spatially-explicit data for simulating earth system dynamics and anthropogenic impacts on the environment.

Compared with existing global LULC datasets for future periods, our dataset gave full account to the variation in future suitability probability relative to its historical counterpart, and employed future driving factors to derive updated suitability probability for LULC expansion across different SSP-RCP scenarios. Moreover, we used the PLUS model, a CA model that is capable of modeling the expansion of multiple types of natural LULC patches, to iteratively simulate the LULC change across different water-basin regions at regular intervals. This iterative approach fully considers the dynamic influence of the prior period’s LULC on the spatial distribution in subsequent periods. By controlling the number of changed pixels, we aimed to ensure that the LULC expansion is more reflective of the actual conditions. This may well demonstrate the empirical dynamics of natural land patch transformations and enable simulations on a fine scale. In addition, the LULC demand for each region at the water-basin level across different SSP-RCP scenarios is generated from the GCAM model, which ensures the comparability of the LULC simulation results under all future scenarios.

Yet, this dataset still has some limitations. Compared with the natural LULC types, the correlation coefficient between the simulation results and the observed data for urban areas is relatively lower (see Table 2, approximately 70%). This can be attributed to the complexities involved in simulating the impact of policy implementations on urban development. In addition, the resolution of the ESA-CCI historical LULC data^30^ can reach a spatial detail of 300 m. However, limited by the spatial resolution of driving factor data (see Table 1), the LULC dataset was developed at a 1 km resolution. For future works, given the improvement in the spatially-explicit details of driving factors, we aim to employ high-resolution, multi-type driving factors to enhance the resolution and accuracy of LULC simulations for future scenarios. Meanwhile, in future works we endeavor to update the versions of the dataset by including a broader range of LULC types and SSP-RCP scenarios so as to provide solid data support for modeling earth system dynamics and human-earth interaction.

### Supplementary information

Supplementary Information

## Data Availability

The computing codes of the GCAM model along with parameter settings are available at https://github.com/JGCRI/gcam-core. The interface software of the PLUS model can be obtained from https://github.com/HPSCIL/Patch-generating_Land_Use_Simulation_Model.
