# Supplementary material for: Mapping the spatial heterogeneity of global land use and land cover from 2020 to 2100 at a 1 km resolution
Source: Sci Data. 2023 Oct 28;10:748. doi: 10.1038/s41597-023-02637-7 (PMC10613310; doi:10.1038/s41597-023-02637-7)
Supplement: Supplementary file 1 — Supplementary Information [file 41597_2023_2637_MOESM1_ESM.docx]

**Supplementary Information of**

**Mapping the spatial heterogeneity of global land use and land cover from 2020 to 2100 at a 1km resolution**

Tianyuan Zhang^1^, Changxiu Cheng^1,4^, Xudong Wu^2,3^

1. State Key Laboratory of Earth Surface Processes and Resource Ecology, Beijing Normal University, Beijing, 100875, PR China

2. School of Soil and Water Conservation, Beijing Forestry University, Beijing, 100083, PR China

3. Research Department of Complexity Science, Potsdam Institute for Climate Impact Research, Potsdam, 14473, Germany

4. National Tibetan Plateau Data Center, Beijing, 100101, PR China

corresponding author(s): Changxiu Cheng ([chengcx@bnu.edu.cn](mailto:chengcx@bnu.edu.cn)), Xudong Wu ([wuxudong@bjfu.edu.cn](mailto:wuxudong@bjfu.edu.cn))

**Table of Contents**

**Texts**

Text S1. Parameter calibration of the PLUS model. …………………………………………………………… 3

Text S2. Metrics for accuracy assessment. …………………………………………………………………………. 3

Text S3. The dominant factors for the expansion of each LULC type on a global scale. ……… 4

Text S4. Global LULC change from 2020 to 2100 under representative SSP-RCP scenarios. … 4

**Figures**

Figure S1. Distribution of global regions at the water-basin level under the GCAM model. … 5

Figure S2. The schematic diagrams of the dataset produced in this study. ………………….…….…. 6

Figure S3. The spatial distribution of Figure of Merit (FoM) values in each water-basin region.

………..………………………………………………………………………………………………………………………………… 6

Figure S4. The spatial distribution of Kappa coefficient values in each water-basin region. … 7

Figure S5. The spatial distribution of overall accuracy (OA) values in each water-basin region.

……………………………………………………………………………………………………………………………………………. 7

Figure S6. Historical spatial distribution maps of each driving factor. …………………………………. 8

Figure S7. Global average contribution rate of driving factors to the expansion of each LULC type. ……………………………………………………………………………………………………………………………… 9

Figure S8. The spatial distribution of dominant driving factors of forest expansion in each

water-basin region. ……………………….…………………………………………………………………………………… 10

Figure S9. The spatial distribution of dominant driving factors of cropland expansion in each

water-basin region. ……….…………………………………………………………………………………………………… 10

Figure S10. The spatial distribution of dominant driving factors of grassland expansion in

each water-basin region. .………………………………………………………………………………………………….. 11

Figure S11. The spatial distribution of dominant driving factors of urban expansion in each water-basin region. .………………………………………………………………………………………………………….. 11

Figure S12. The spatial distribution of dominant driving factors of barren expansion in each water-basin region. …………………………………………………………………………………………………………... 12

Figure S13. The spatial distribution of dominant driving factors of water expansion in each water-basin region. …………………………………………………………………………………………………………... 12

Figure S14. The spatial distribution of the LULC changes from 2020 to 2100 under different SSP-RCP scenarios. ……………………………………………………………………………………………………………. 13

Figure S15. Distribution of LULC in the case region of Southeast Asia for 2020 (a) and 2100 (future scenarios, b–f). ……………………………………………………………………………………………………... 14

Figure S16. Distribution of LULC in the case region of South America for 2020 (a) and 2100 (future scenarios, b–f). ……………………………………………………………………………………………………... 15

Figure S17. Distribution of LULC in the case region of Tibetan Plateau for 2020 (a) and 2100 (future scenarios, b–f). ……………………………………………………………………………………………………... 16

Figure S18. Comparison of the simulated LULC results of a typical case region (South Africa) in the years 2030, 2050, 2070 and 2100 under the SSP2-4.5 scenario between the dataset

produced in this study and the Global LULC dataset. ………………………………………………………..... 17

**Tables**

Table S1. LULC classification and mapping relationships among different products. .…………. 18

Table S2. The FoM, Kappa coefficient, and OA values for each water-basin region. ………....... 20

**References** ..………………………………………………………………………………………………………………………. 23

### Texts

**Text S1. Parameter calibration of the PLUS model****.**

Parameter calibration for CA models during the process of LULC simulation is crucial to achieve better simulation results. There are two categories of adjustable parameters in the PLUS model. (1) Parameters related to random forest modeling in the LEAS module, including the sampling method (random or uniform sampling), the number of regression trees, and the sampling rate. (2) Parameters for patch morphology regulation in the CARS Module, including neighborhood size, patch generation threshold, expansion coefficient, and percentage of seeds. The patch generation threshold represents the descending threshold for generating new patches, ranging from 0 to 1. A higher threshold implies a more conservative transformation strategy, reducing the likelihood of changes in cells with lower suitability probabilities. The expansion coefficient adjusts the model’s capability to generate new LULC patches, with a range of 0 to 1. A higher expansion coefficient indicates a greater ability to generate new patches. The percentage of seeds determines the maximum threshold for generating new seed quantities, ranging from 0 to 1. A higher percentage of seeds results in a more scattered LULC pattern.

We applied the method of controlling variables to determine the values of the aforementioned seven parameters. When adjusting each parameter, we systematically modified their values from the smallest to the largest. Throughout this process, the values of the other six parameters remained constant. We assessed the simulation accuracy after each adjustment, and the value corresponding to the optimal simulation accuracy was selected as the final value for the parameter.

**Text S2. Metrics for** **accuracy assessment.**

In this study, we use Overall Accuracy (OA), Kappa coefficient, and Figure of Merit (FoM) as three metrics to evaluate the accuracy of LULC simulation.

OA refers to the ratio of correctly classified land cells to the total number of cells. The values of OA range between 0 and 1, with a higher value indicating better simulation performance. The formula is as follows:

$$\begin{aligned} OA=\frac{TP+TN}{TP+TN+FP+FN}\#\left( 1 \right) \end{aligned}$$

where TP (True Positive) indicates the number of land cells correctly identified as the target LULC type; TN (True Negative) represents the number of cells that are accurately classified as non-target LULC types; FP (False Positive) refers to the number of cells that are incorrectly classified as the target LULC class; and FN (False Negative) indicates the number of cells that are wrongly allocated into non-target LULC class.

Cohen’s Kappa coefficient is calculated based on the OA. It is employed to further verify whether the simulated results of the land use simulation model are consistent with the actual land use pattern. A Kappa coefficient approaching 1 indicates a higher level of reliability for the simulated results. The formula is as follows:

$$\begin{aligned} K=\frac{P_{0}-P_{e}}{1-P_{e}}\#\left( 2 \right) \end{aligned}$$

where

$$\begin{aligned} P_{0}=OA, \#\left( 3 \right) \end{aligned}$$

$$\begin{aligned} P_{e}=\frac{\left( TP+FN \right)\times\left( TP+FP \right)+\left( FN+TN \right)\times\left( TN+FP \right)}{N^{2}}\#\left( 4 \right) \end{aligned}$$

FoM denotes the proportion of accurately predicted LULC changes in relation to the aggregate of observed and predicted changes. In contrast to the Kappa coefficient, FoM can effectively avoid the overestimation of accuracy and has been demonstrated efficient in the assessment of LULC change simulations^1,2^. A higher FoM value indicates a better simulation accuracy. The formulation of FoM can be expressed as follows:

$$\begin{aligned} FoM=\frac{B}{A+B+C+D}\#\left( 5 \right) \end{aligned}$$

where A represents the count of LULC cells that are observed as changed but predicted as unchanged; B indicates the count of cells that are accurately predicted as changed into the correct LULC class; C represents the count of cells that are predicted as changed into an incorrect LULC category; and D represents the count of cells that are observed as unchanged but predicted as changed^1–3^.

**Text S3. The dominant factors for the expansion of each LULC type on a global scale.**

The sampling strategy employed in simulating land use helps to identify change characteristics in land use/land cover (LULC). These characteristics are combined with the random forest model to generate LULC suitability probabilities. This process enables a suitable measurement of the contribution rates of different driving factors to the expansion of each LULC type. As a result, we can obtain the contribution rate of each driving factor at the water-basin level regions and calculate the global-scale contribution rate by averaging the values across all water-basin regions (see Figure S7).

At the global scale, altitude plays a crucial role in the expansion of cropland, forest, and grassland. However, its contribution rate differs by no more than 6% as compared to other factors (see Figure S7a–c). Regarding the magnitude of urban development, it is primarily determined by socio-economic factors including GDP, population, and administrative centers’ influence (see Figure S7d).

At the water-basin scale, we assess and rank the factors that influence the expansion of each LULC type based on their contribution rates. The dominant factor responsible for the expansion of each LULC type is determined by identifying the factor with the highest contribution rate (see Figure S8–S13).

**Text S4. Global LULC change from 2020 to 2100 under representative SSP-RCP scenarios.**

The simulated global LULC changes from 2020 to 2100 under representative SSP-RCP scenarios are presented in Figure S14. The cropland area in the United States, Eastern Brazil, Europe, Africa, and China is projected to decrease by 0–30% per 100 km^2^. In comparison, the cropland area in the Middle East, Southeast Asia, and Central South America is projected to increase (see Figure S14a). Regarding China’s cropland, it is observed that the reduction will be more evident in the SSP1-2.6 (see Figure S14a1) and SSP4-3.4 (see Figure S14a4) scenarios, while the SSP5-8.5 (see Figure S14a5) scenario features the smallest decrease. Additionally, under the SSP4-3.4 (see Figure S14a4) scenario, the reduction in cropland area is comparatively marginal for America and Europe, while Africa substantially increases cropland area.

The expansion of forested regions (see Figure S14b) is projected to be most prominent in the SSP1-2.6 (see Figure S14b1) and SSP2-4.5 (see Figure S14b2) scenarios, which are concentrated in Europe, the Russian Far East, and Northeast China. Meanwhile, a notable upward trend in the forest area of North America and Africa is observed across all scenarios.

The grassland area in Canada and the Russian Far East (see Figure S14c) is projected to experience a decline under all scenarios, while the grassland area in Eastern Europe will also decrease except in the SSP4-3.4 scenario (see Figure S14c4). However, the proportion of grassland areas in the United States, Eastern Russia, and South America is expected to increase across all scenarios. Additionally, the grassland area in Africa will expand in the SSP1-2.6 (see Figure S14c1) and SSP2-4.5 (see Figure S14c2) scenarios, and a similar trend will emerge in China under the SSP4-3.4 scenario.

The expansion of urban regions (see Figure S14d) will mainly occur in Western Europe and along the coastal regions of China. The reduction in barren land (see Figure S14e) will be primarily concentrated in Russia’s Eastern and Far Eastern regions. However, under the SSP5-8.5 scenario (depicted in Figure S14e5), it is evident that the barren area in Central Asia will experience a noticeable increase.

### Figures

**
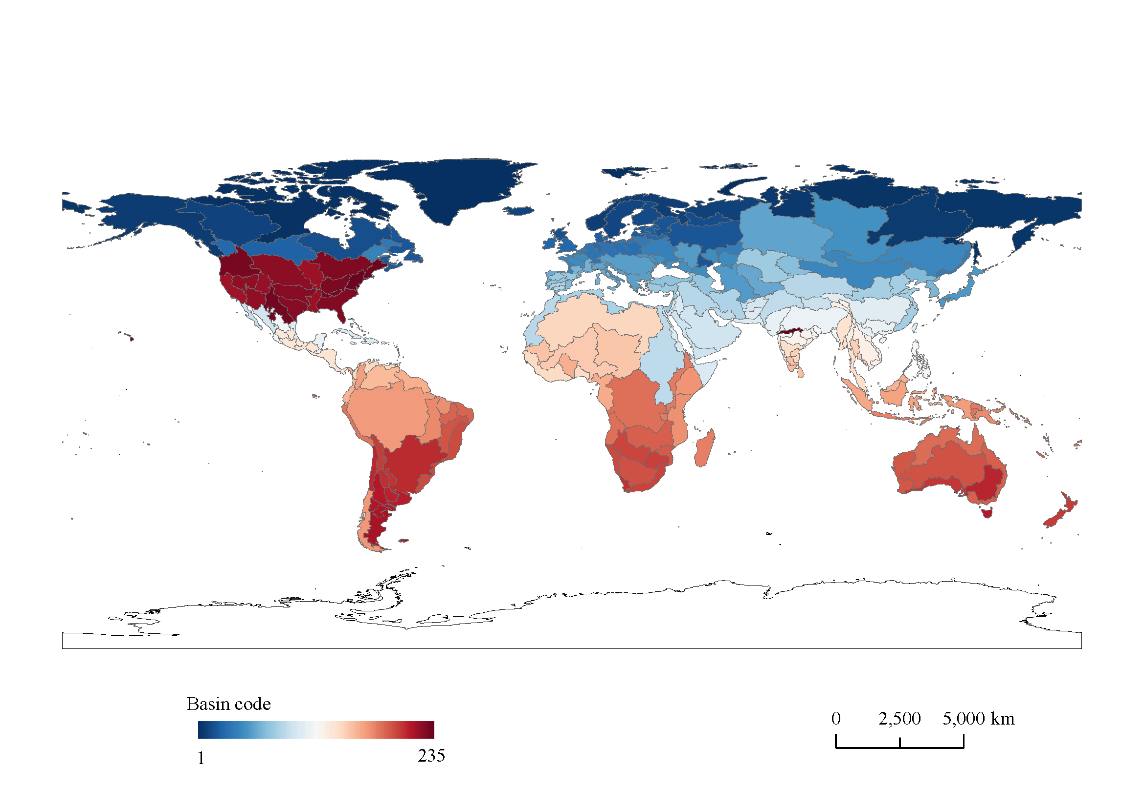
**

Figure S1. Distribution of global regions at the water-basin level under the GCAM model.


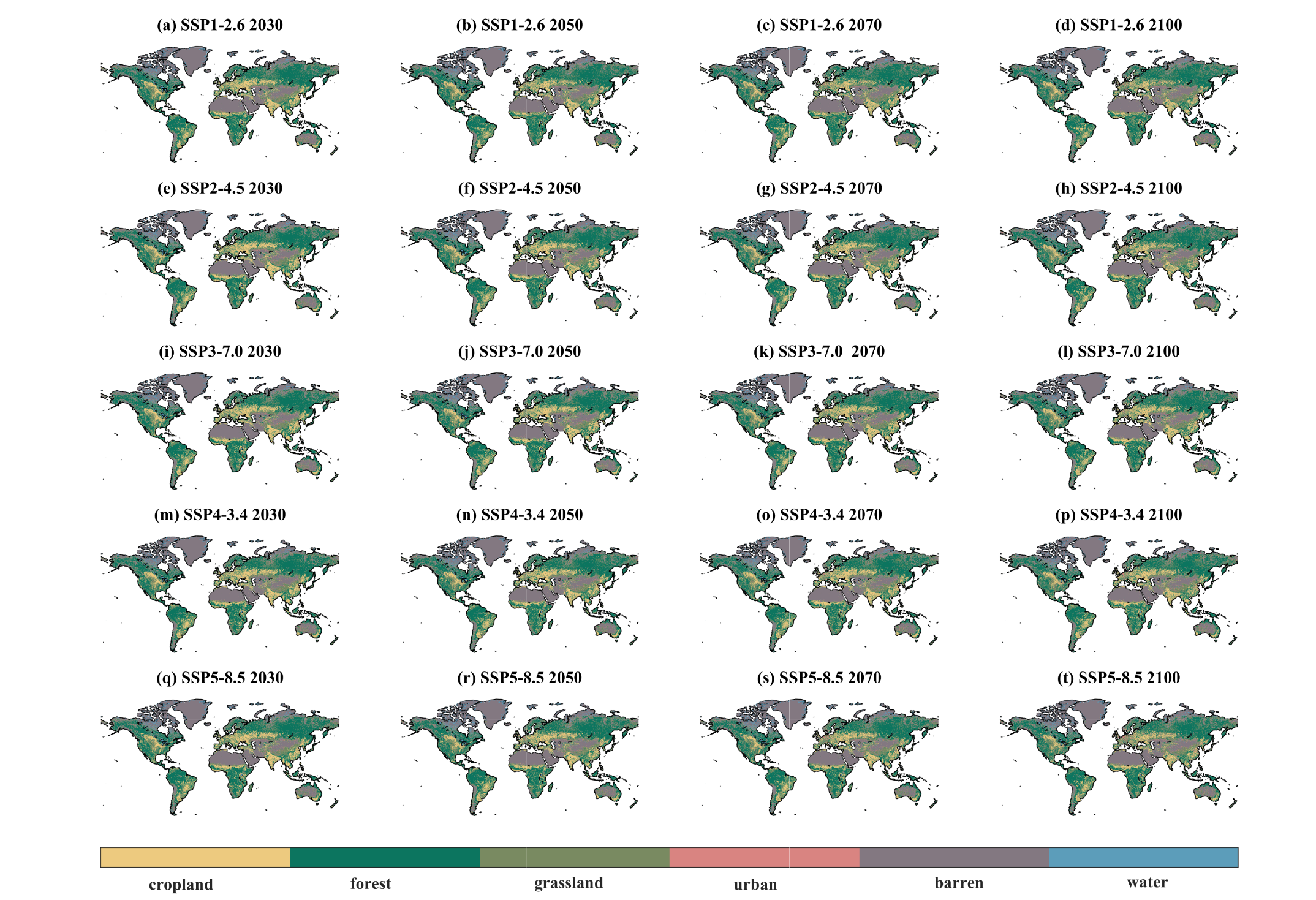


Figure S2. The schematic diagrams of the dataset produced in this study (taking 2030, 2050, 2070 and 2100 as reference years). The schematic diagrams depict the spatial-temporal distribution of global LULC in future scenarios (the dataset was resampled from 1 km to 10 km resolution to create these diagrams).

*
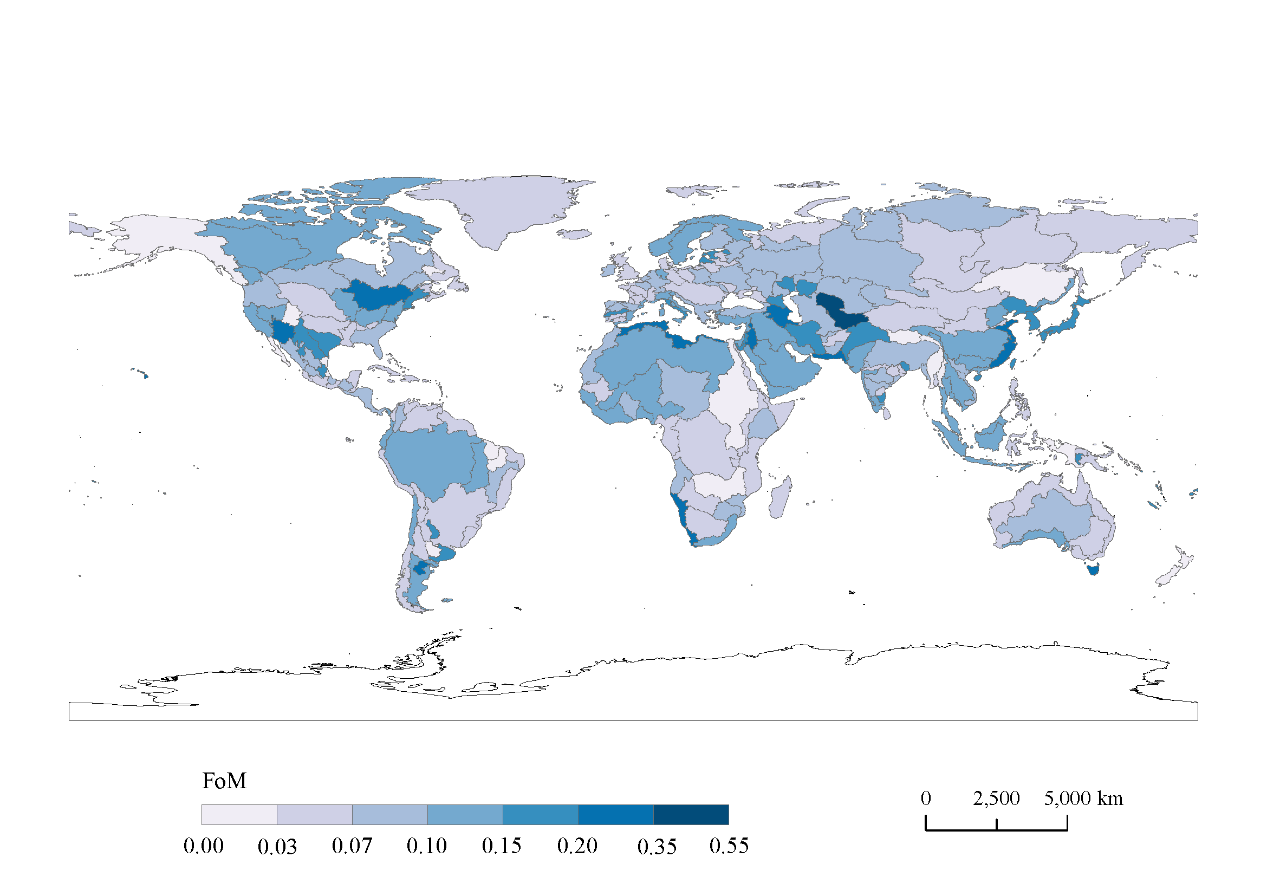
*

Figure S3. The spatial distribution of Figure of Merit (FoM) values in each water-basin region.


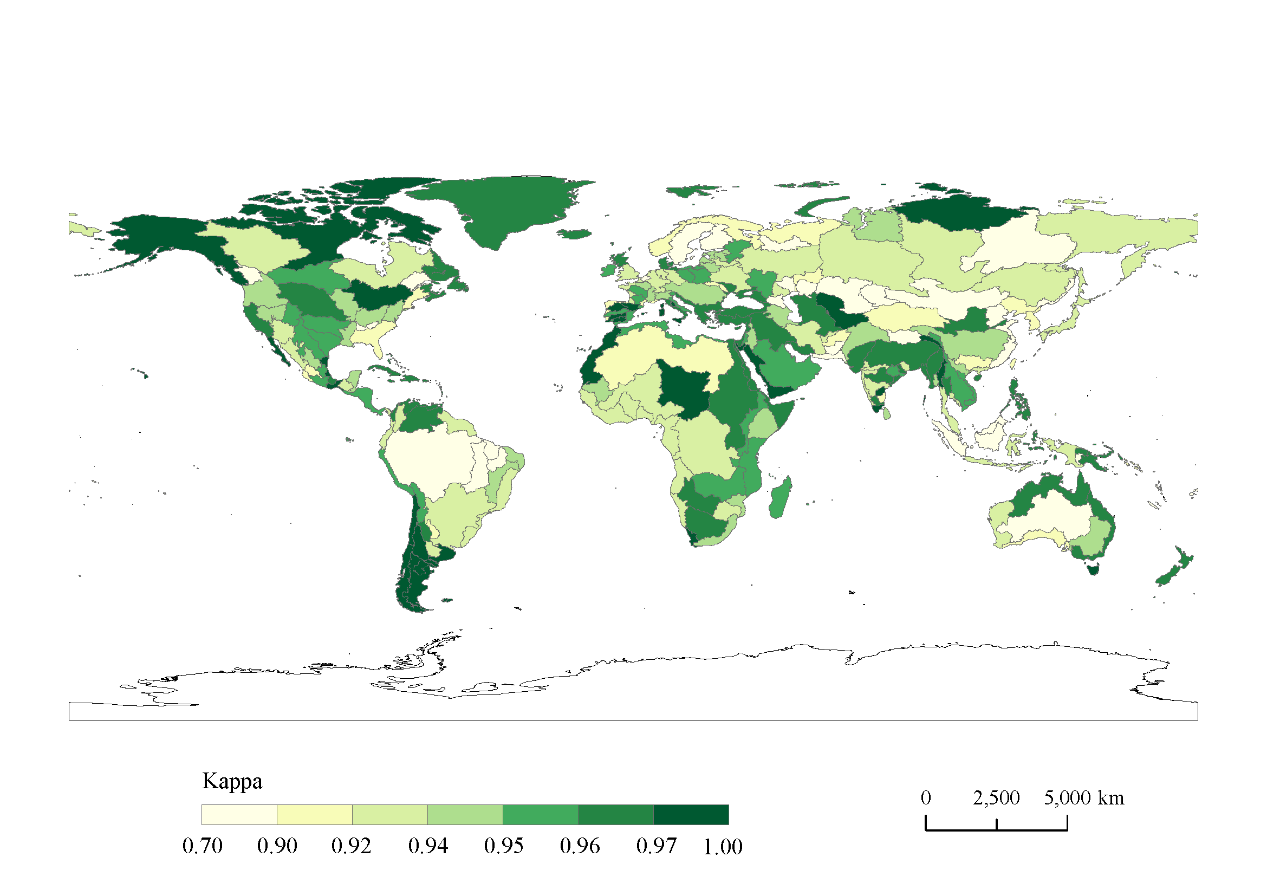


Figure S4. The spatial distribution of Kappa coefficient values in each water-basin region.

*
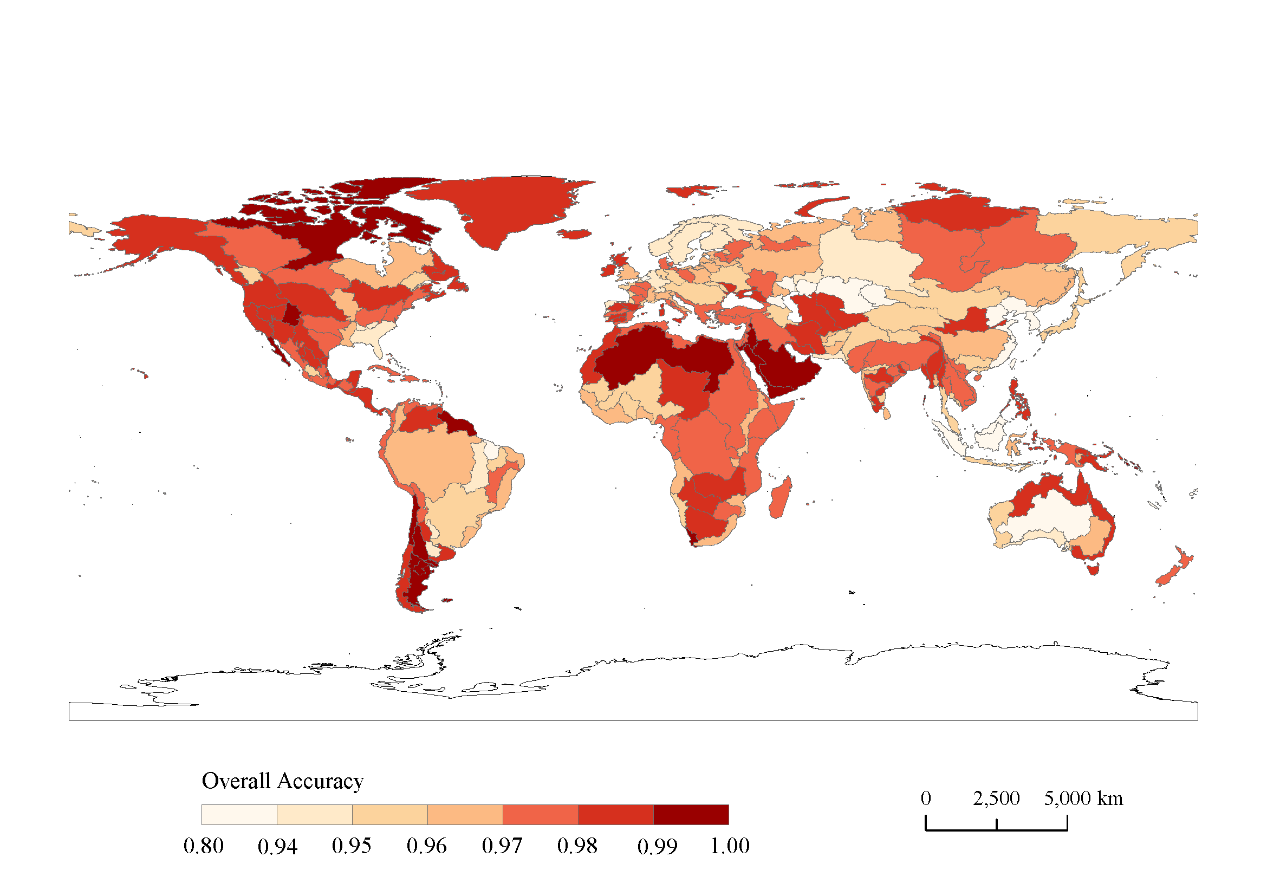
*

Figure S5. The spatial distribution of overall accuracy (OA) values in each water-basin region.


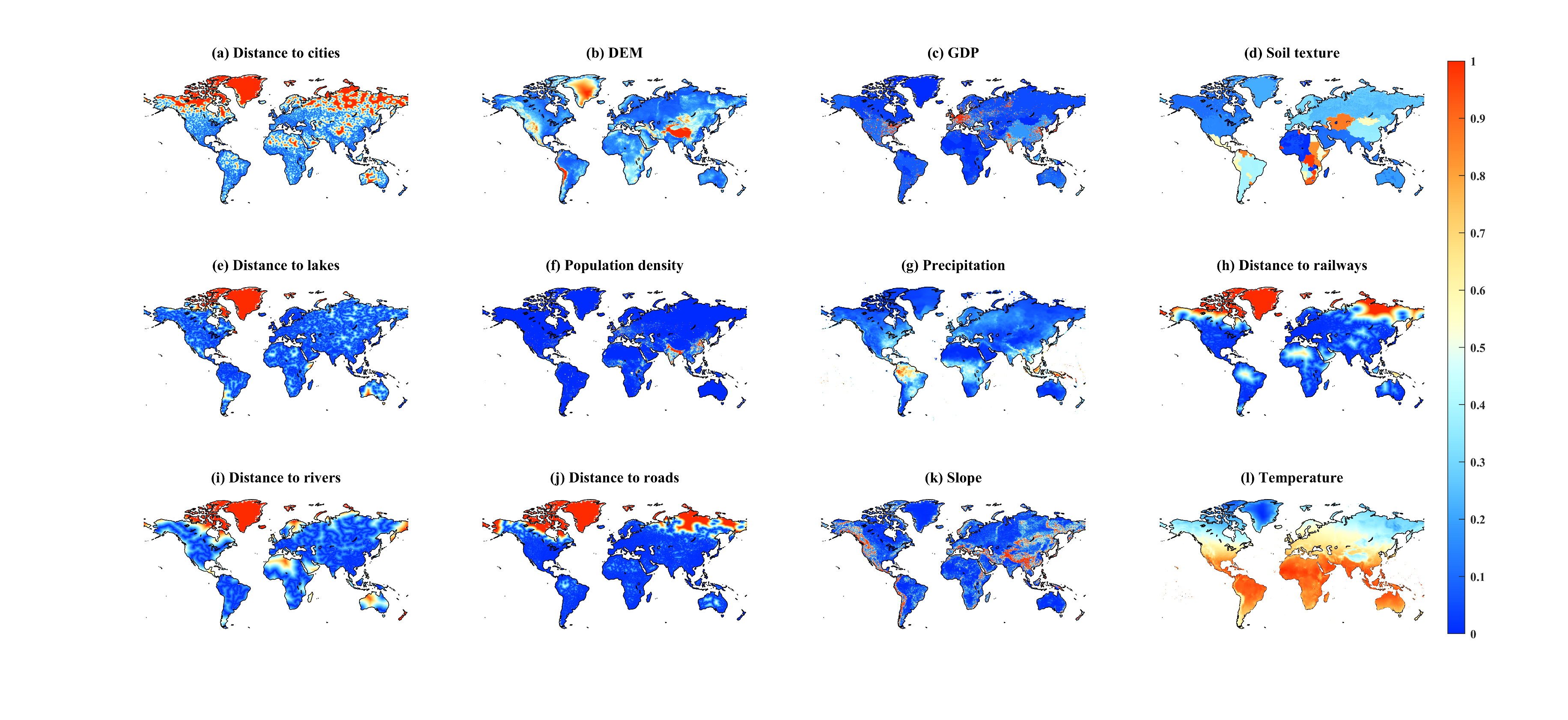


Figure S6. Historical spatial distribution maps of each driving factor.


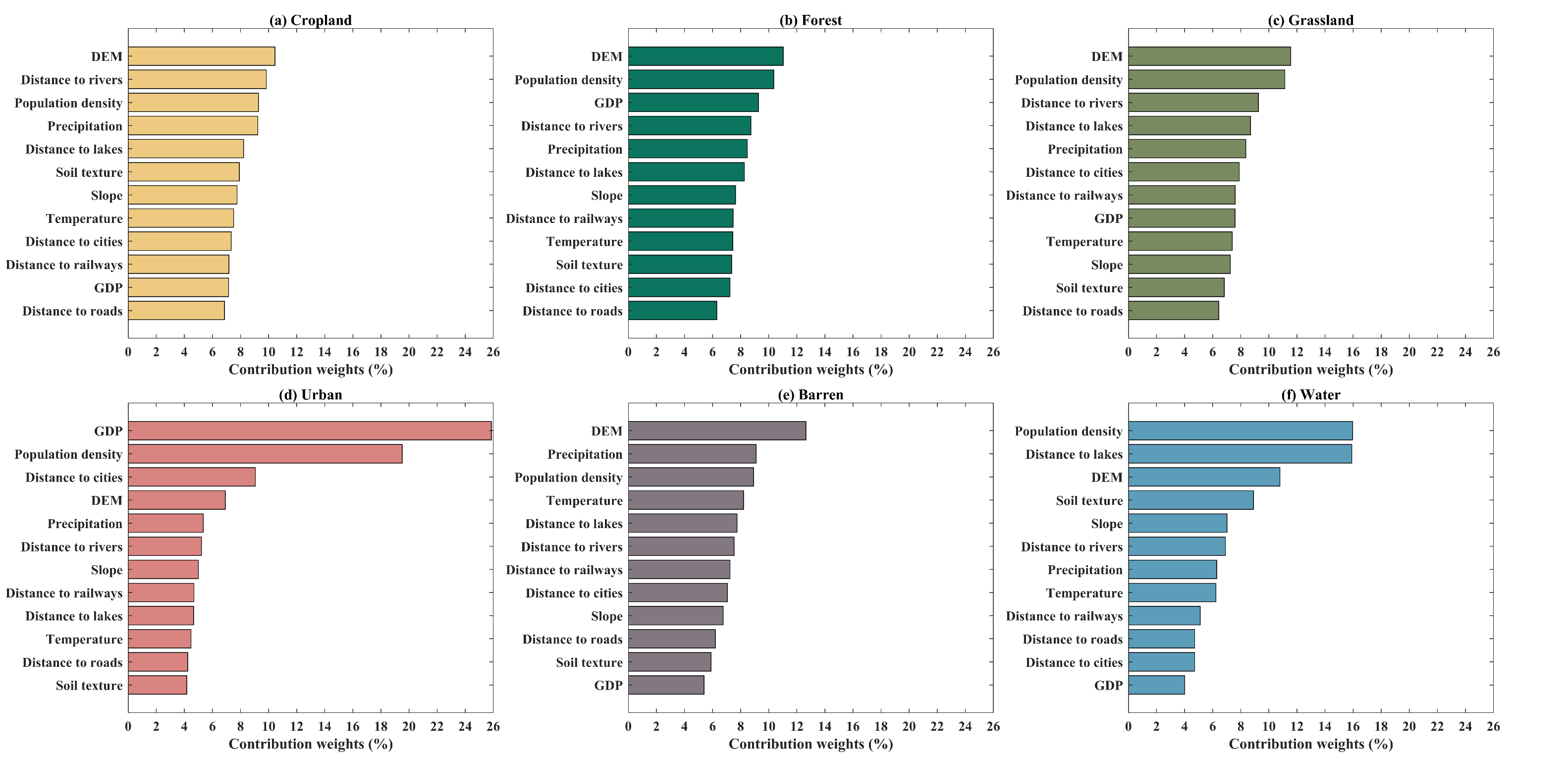


Figure S7. Global average contribution rate of driving factors to the expansion of each LULC type.


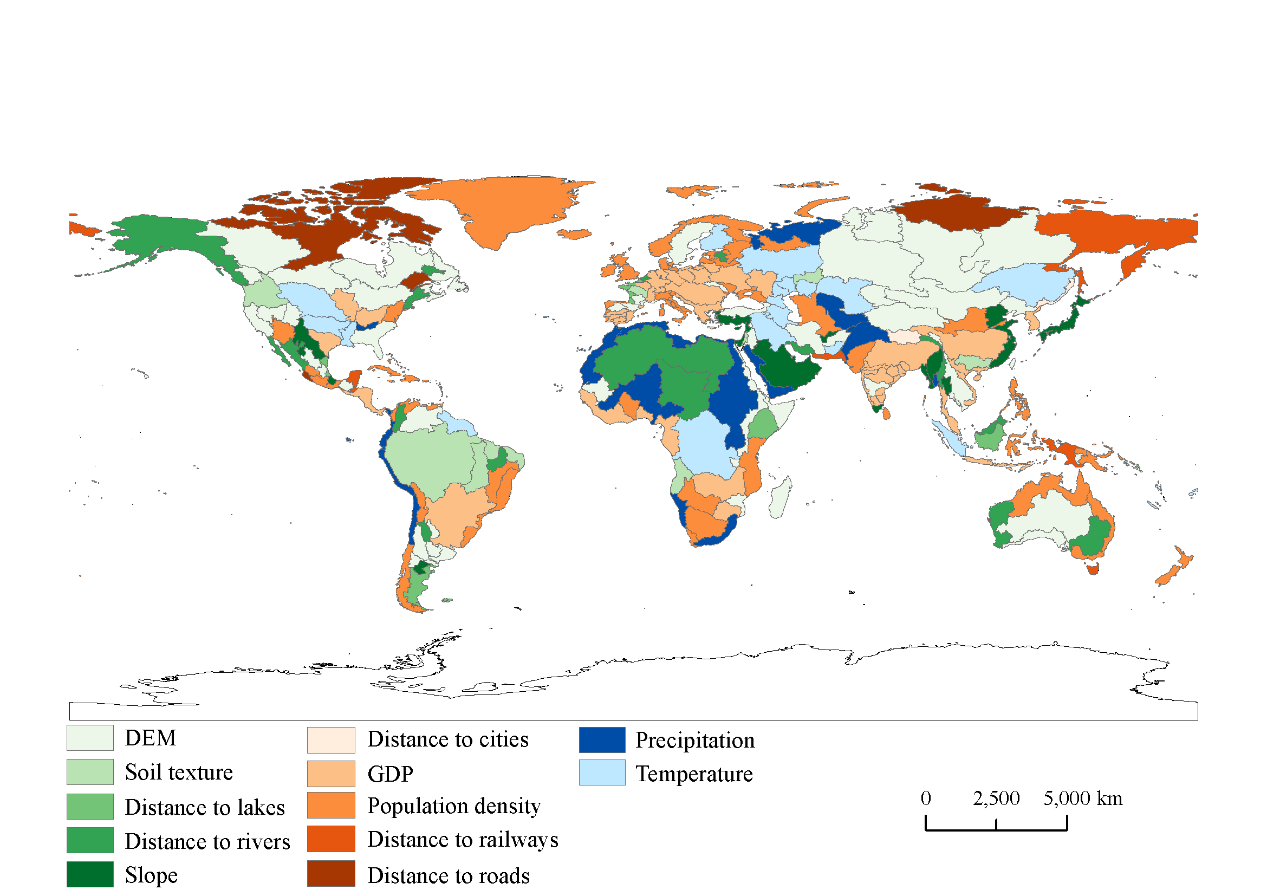


Figure S8. The spatial distribution of dominant driving factors of forest expansion in each water-basin region. The dominant factors are categorized into three types: the physical geographic environment (represented by green filling), socio-economic development (represented by red filling), and climate change (represented by blue filling).


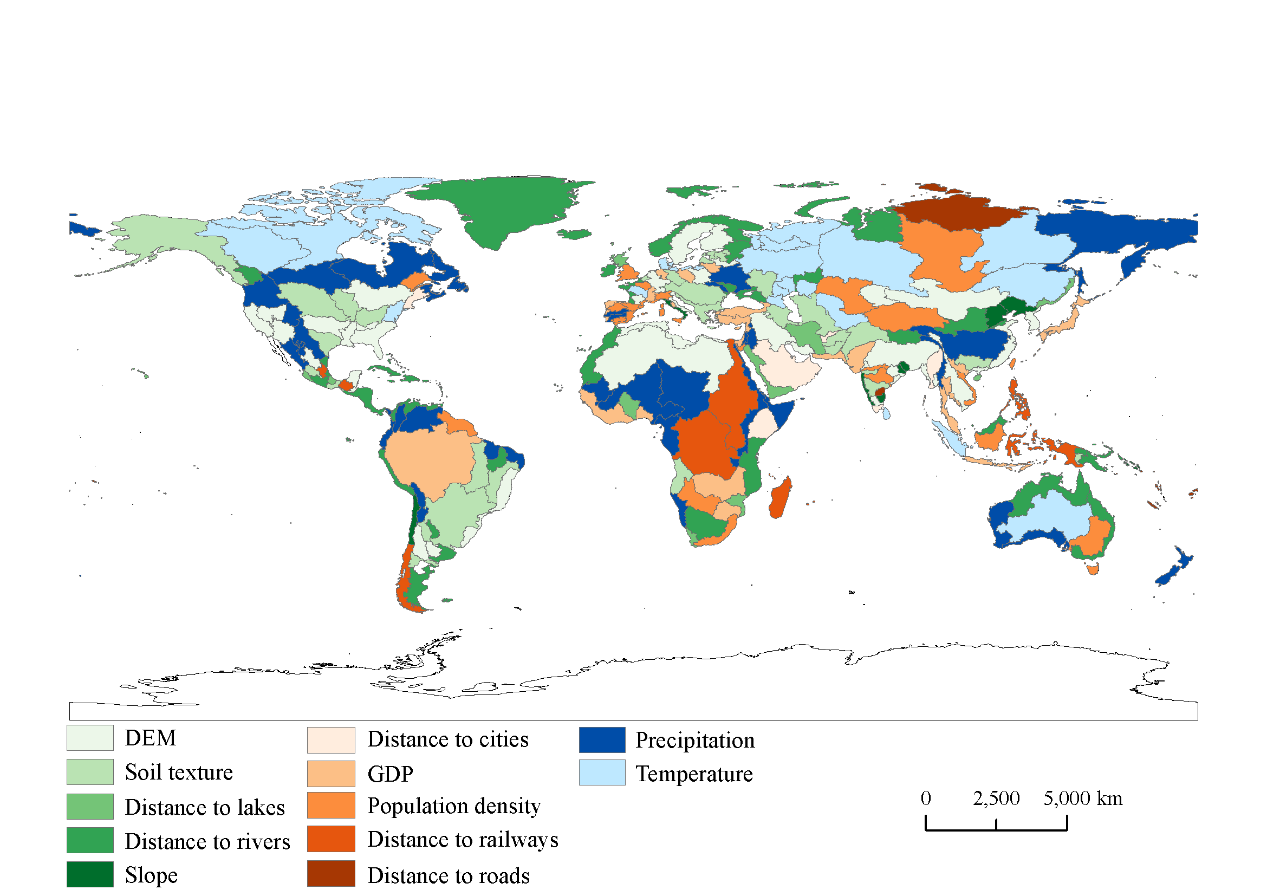


Figure S9. The spatial distribution of dominant driving factors of cropland expansion in each water-basin region. The dominant factors are categorized into three types: the physical geographic environment (represented by green filling), socio-economic development (represented by red filling), and climate change (represented by blue filling).


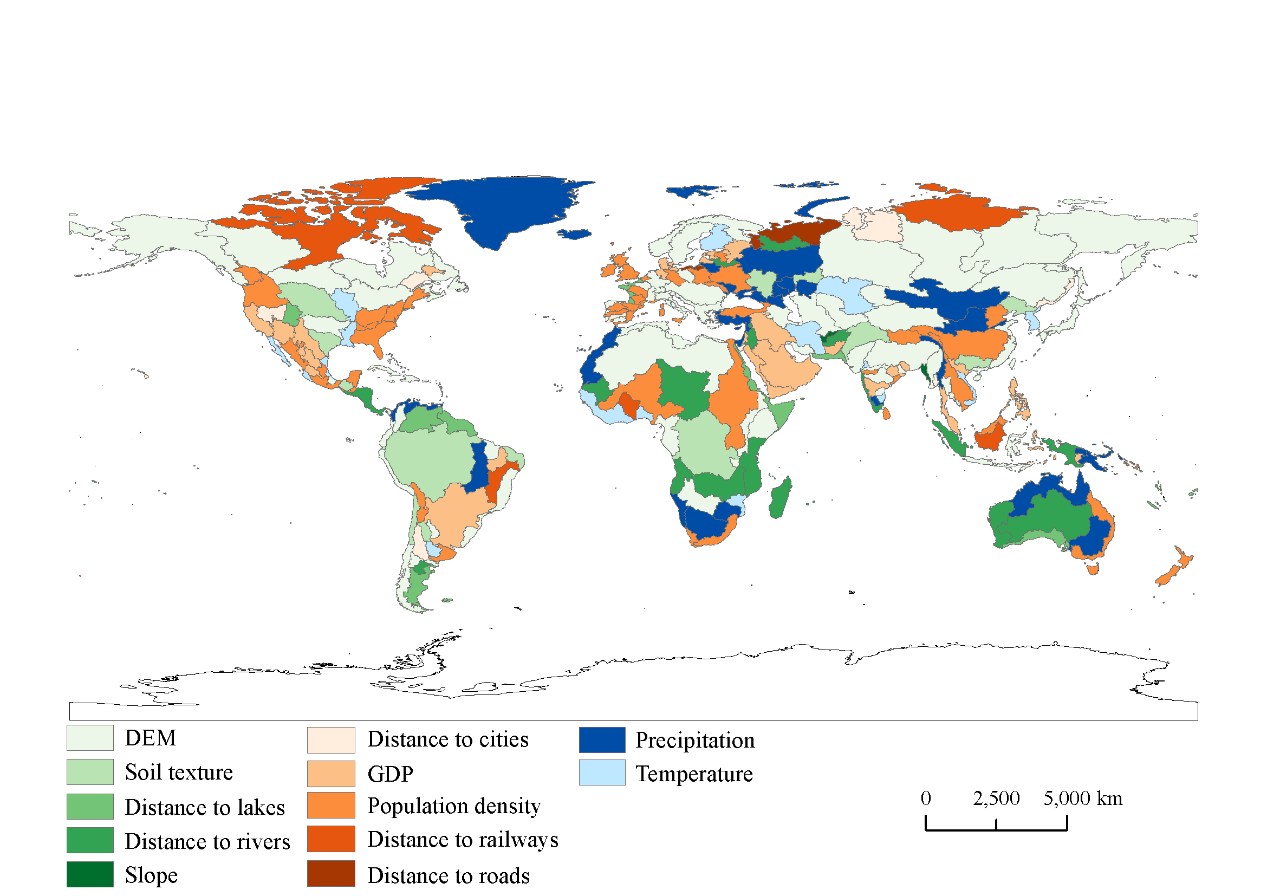


Figure S10. The spatial distribution of dominant driving factors of grassland expansion in each water-basin region. The dominant factors are categorized into three types: the physical geographic environment (represented by green filling), socio-economic development (represented by red filling), and climate change (represented by blue filling).


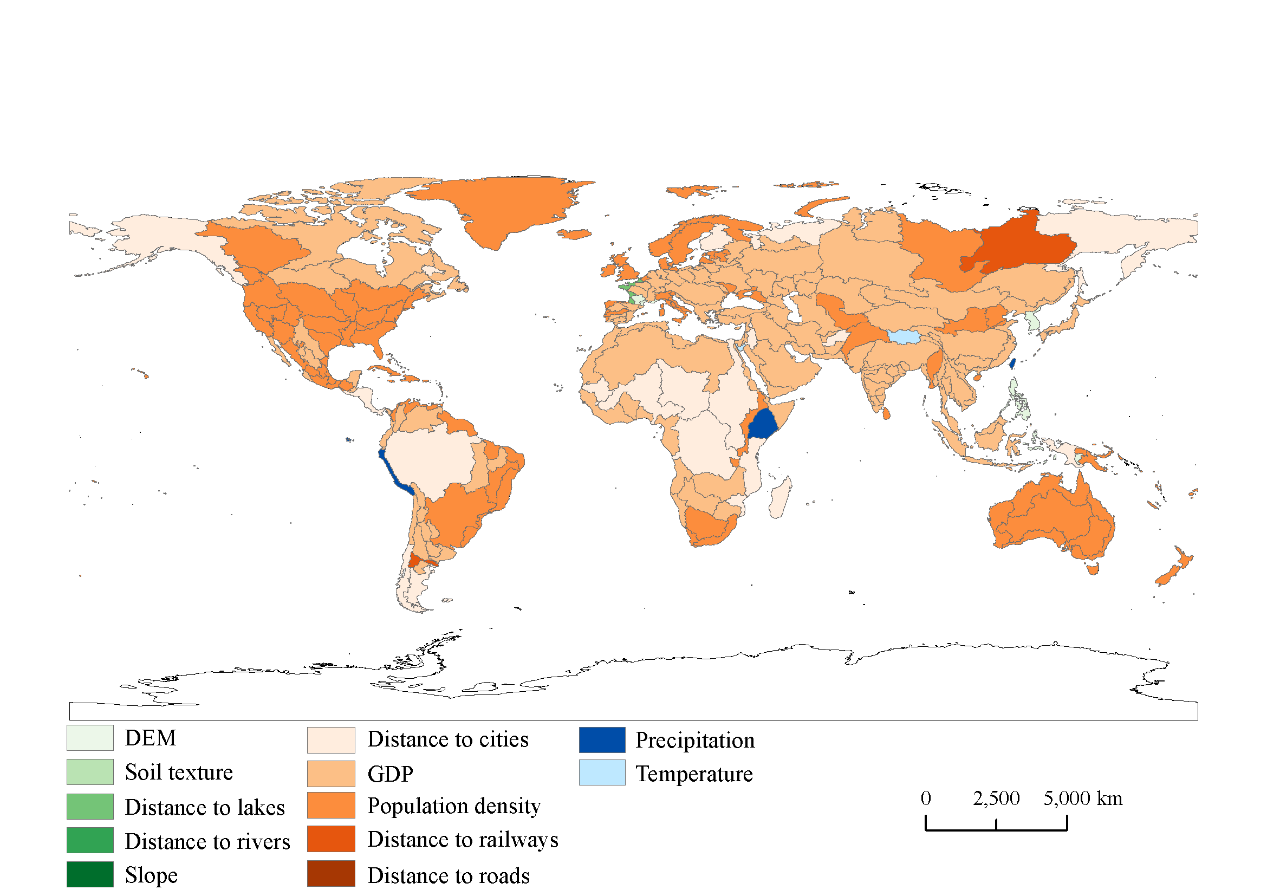


Figure S11. The spatial distribution of dominant driving factors of urban expansion in each water-basin region. The dominant factors are categorized into three types: the physical geographic environment (represented by green filling), socio-economic development (represented by red filling), and climate change (represented by blue filling).


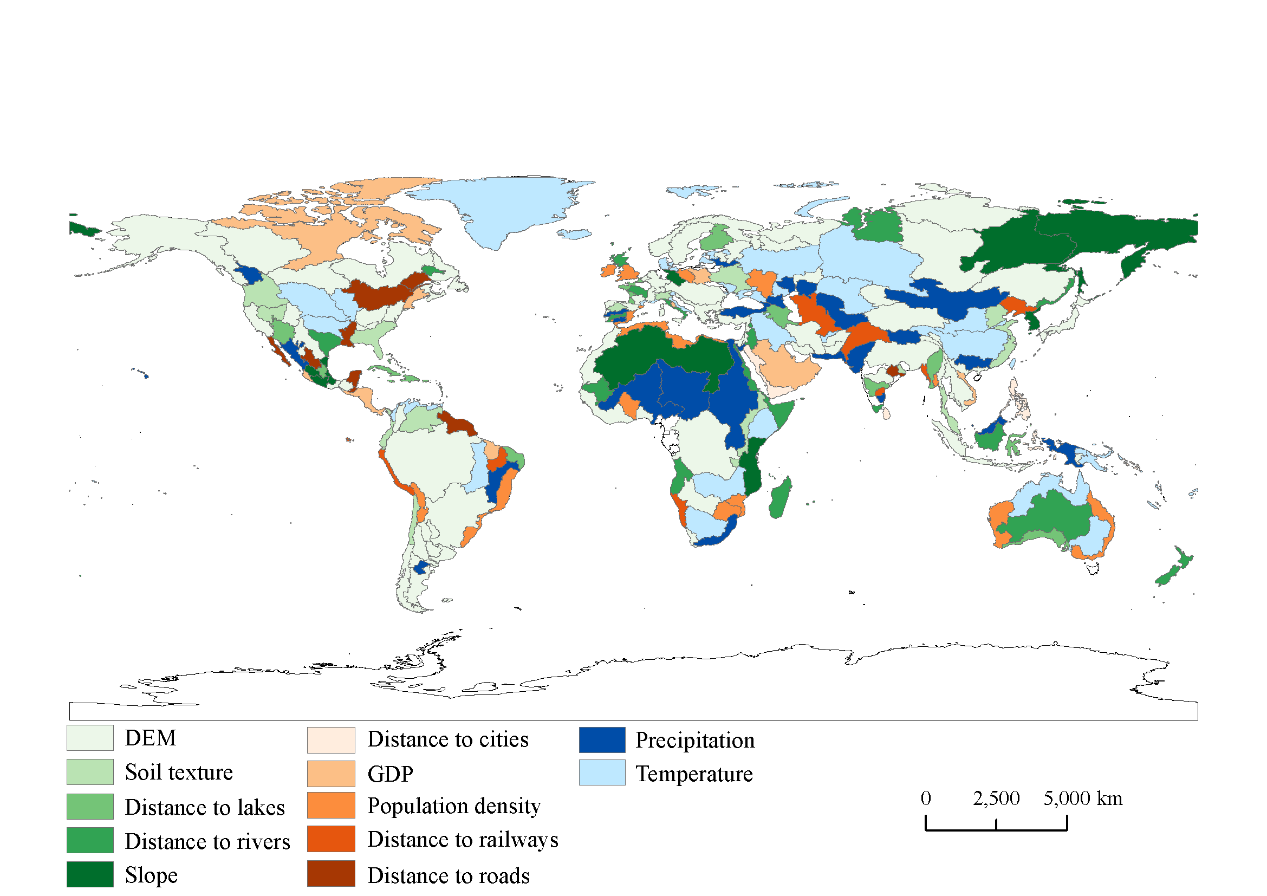


Figure S12. The spatial distribution of dominant driving factors of barren expansion in each water-basin region. The dominant factors are categorized into three types: the physical geographic environment (represented by green filling), socio-economic development (represented by red filling), and climate change (represented by blue filling).


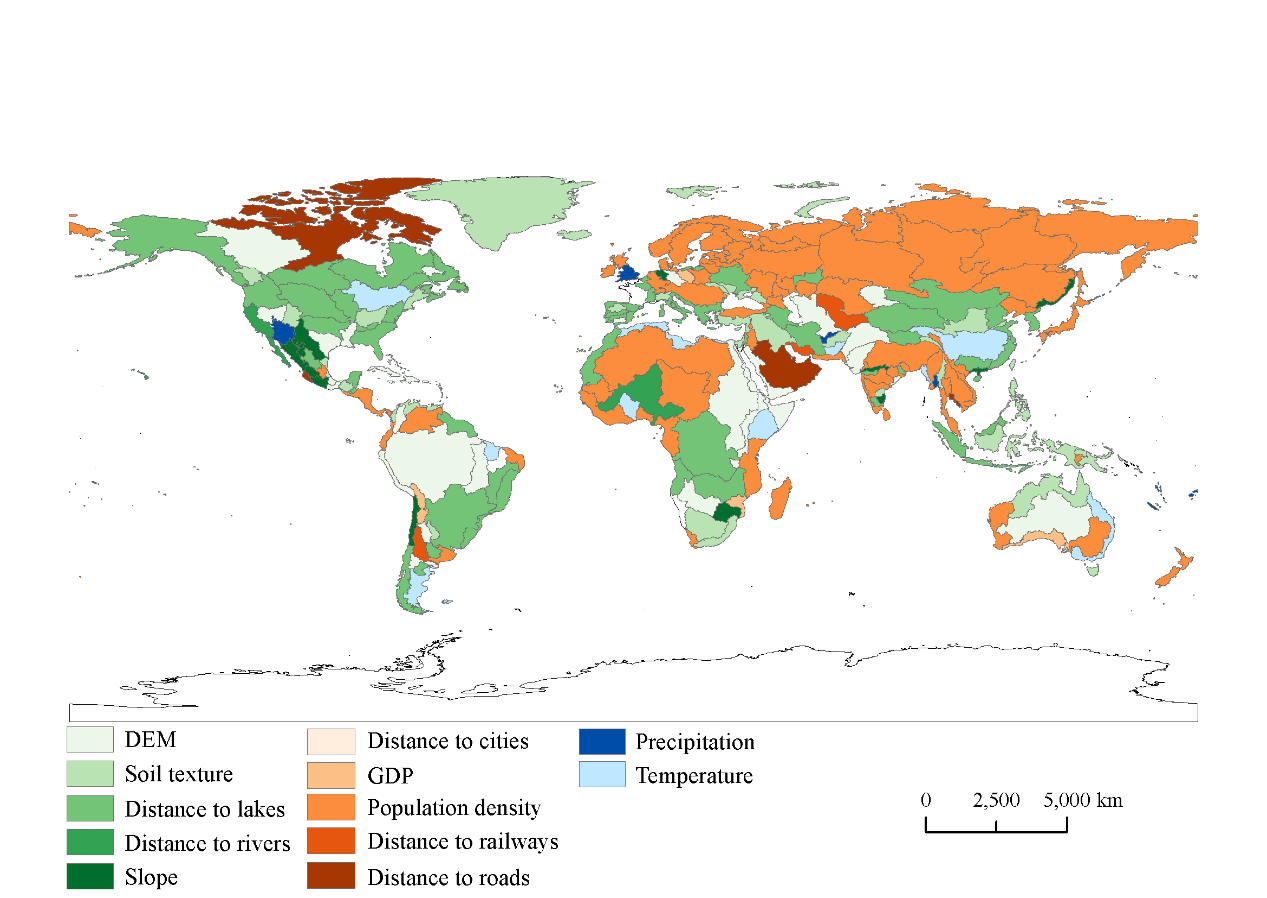


Figure S13. The spatial distribution of dominant driving factors of water expansion in each water-basin region. The dominant factors are categorized into three types: the physical geographic environment (represented by green filling), socio-economic development (represented by red filling), and climate change (represented by blue filling).


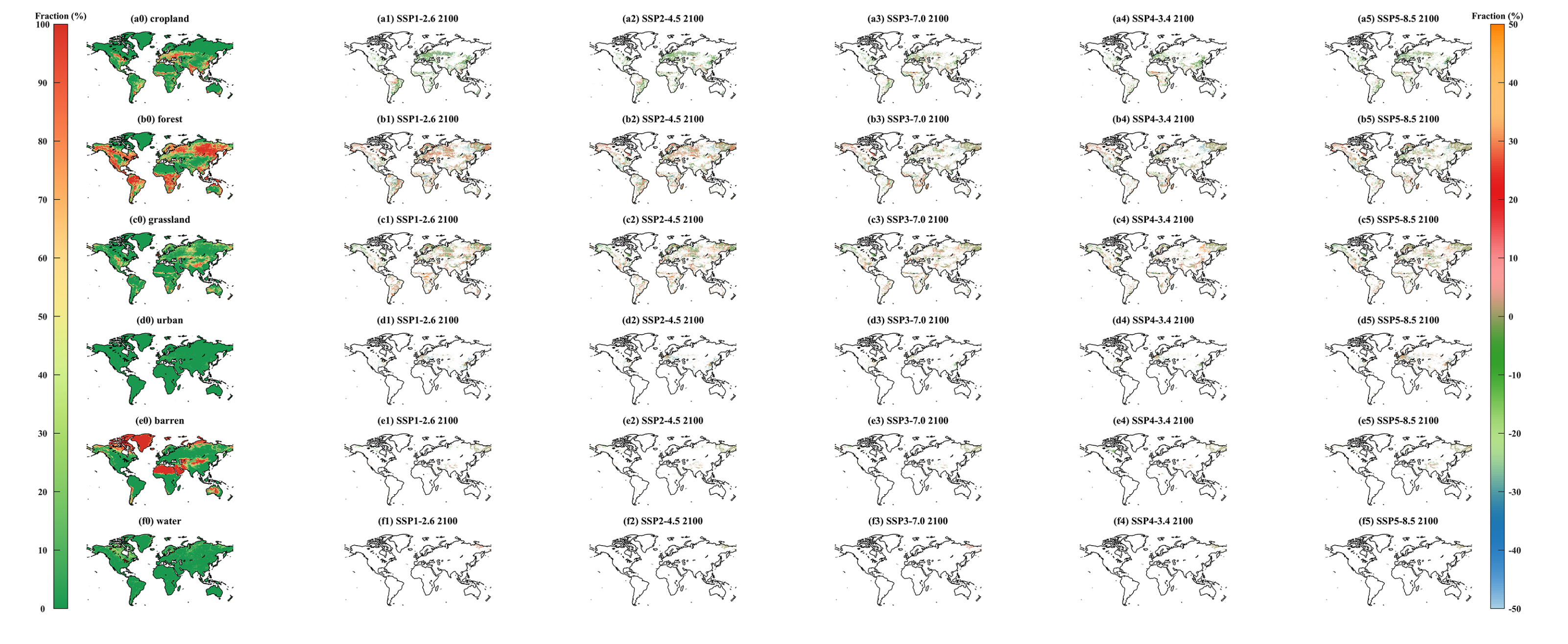


Figure S14. The spatial distribution of the LULC changes from 2020 to 2100 under different SSP-RCP scenarios. Each subplot represents the proportional area of the corresponding LULC type and their changes after aggregating 1 km × 1 km grids into 10 km × 10 km grids. Rows 1 to 6 represent six LULC types: cropland (a), forest (b), grassland (c), urban (d), barren (e) and water (f). The first column represents the historical LULC distribution in 2020, while columns 2 to 6 represent the LULC changes from 2020 to 2100 corresponding to five scenarios: SSP1-2.6, SSP2-4.5, SSP3-7.0, SSP4-3.4, and SSP5-8.5.


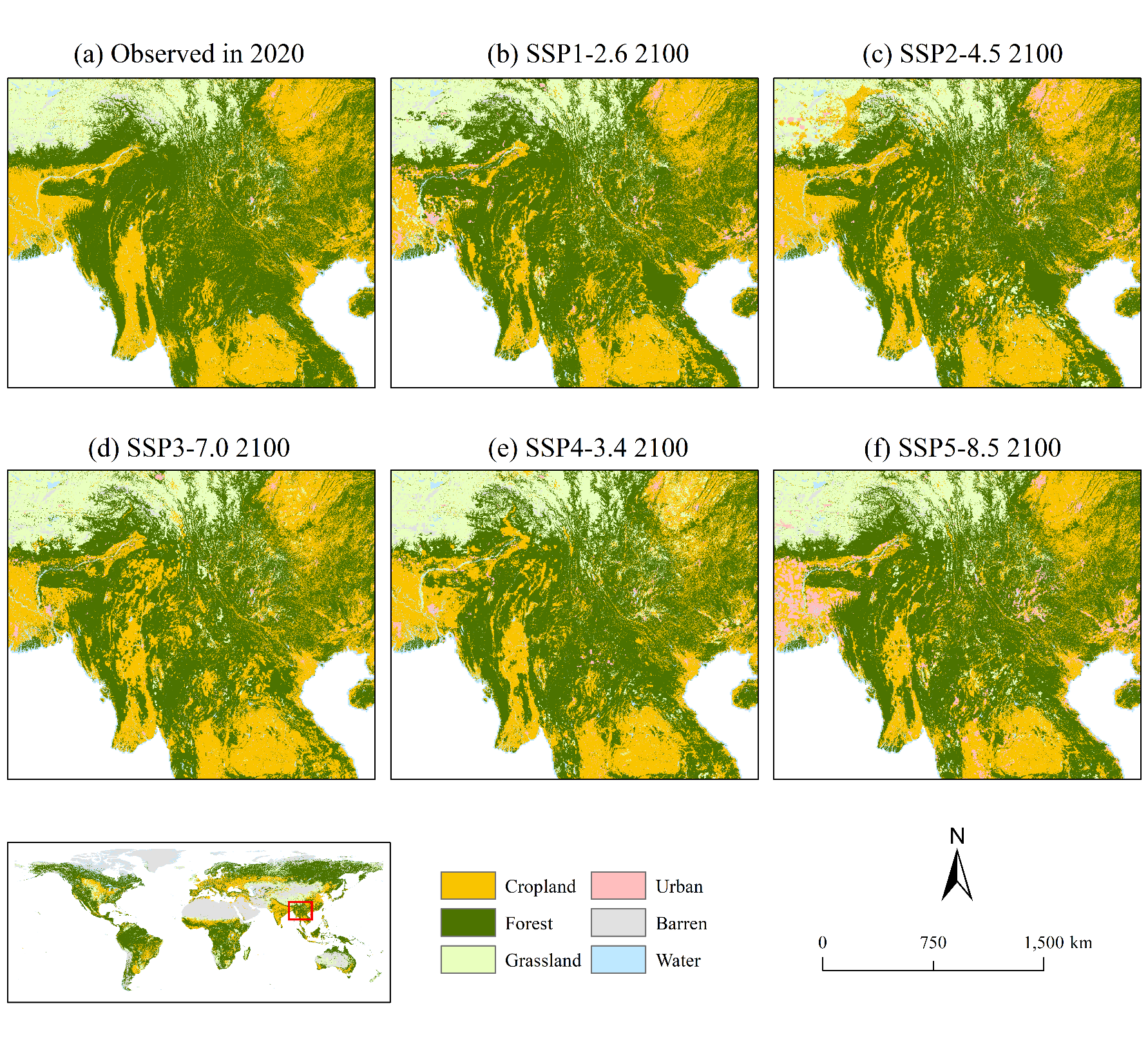


Figure S15. Distribution of LULC in the case region of Southeast Asia for 2020 (a) and 2100 (future scenarios, b–f).


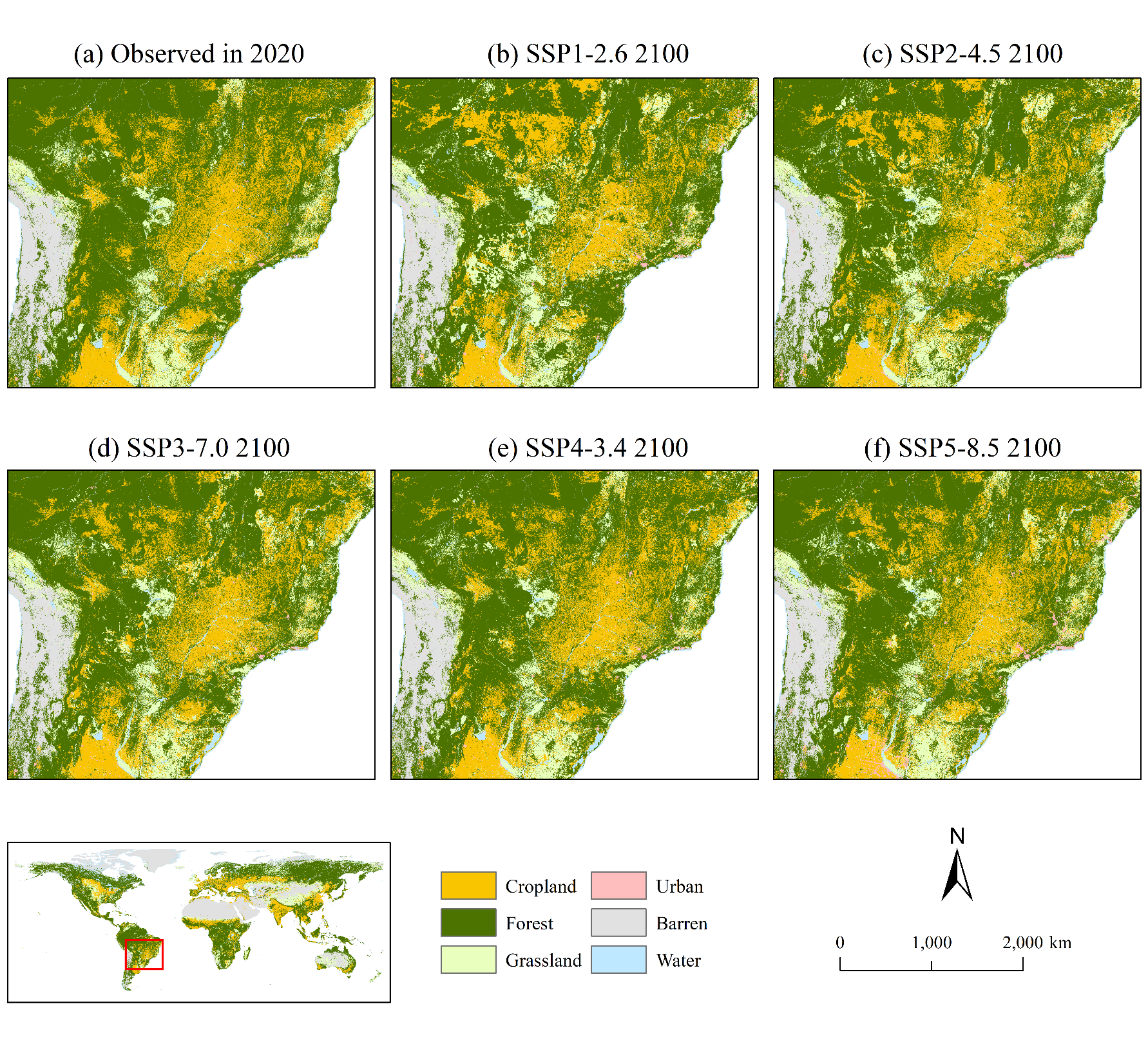


Figure S16. Distribution of LULC in the case region of South America for 2020 (a) and 2100 (future scenarios, b–f).


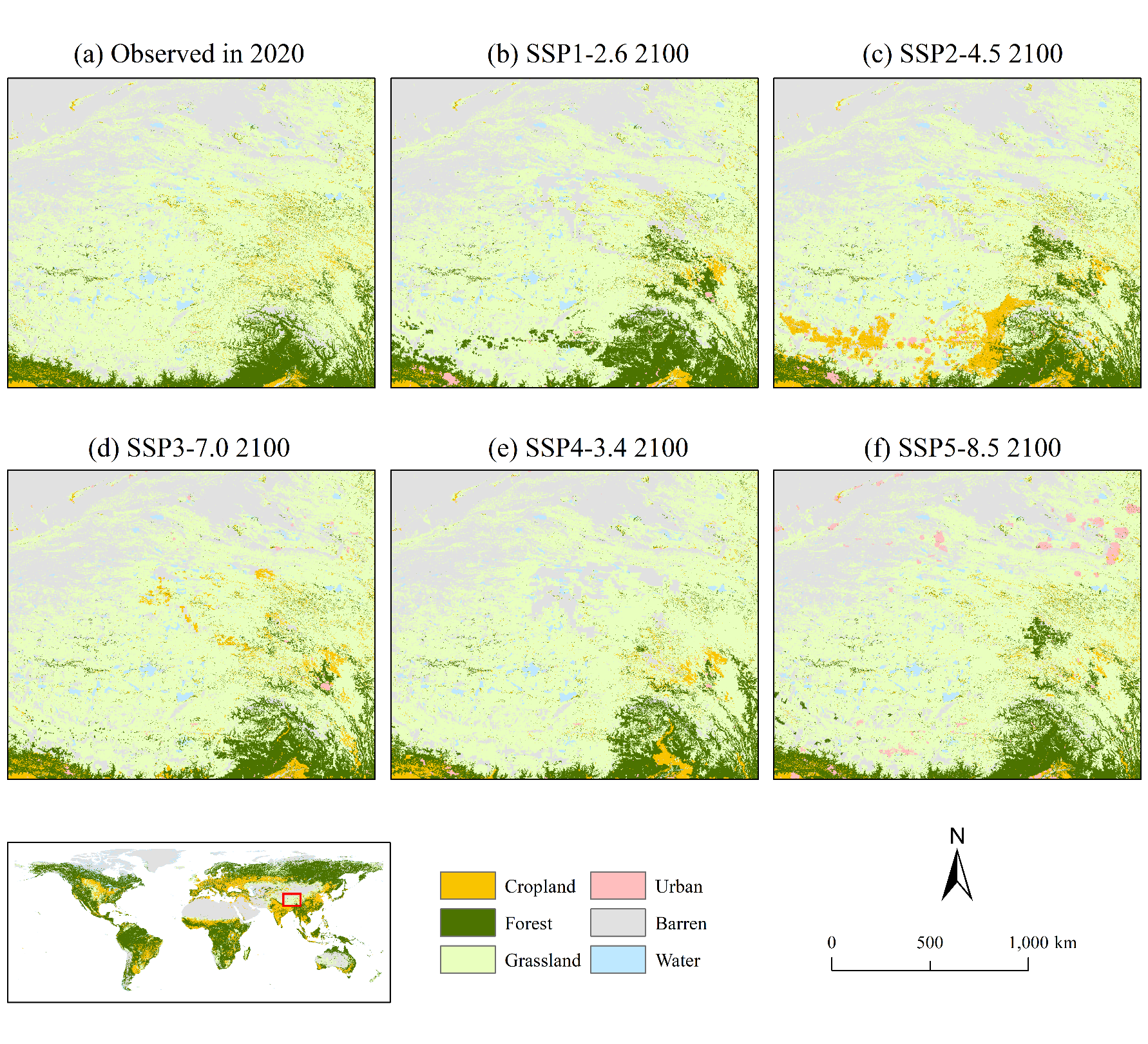


Figure S17. Distribution of LULC in the case region of Tibetan Plateau for 2020 (a) and 2100 (future scenarios, b–f).


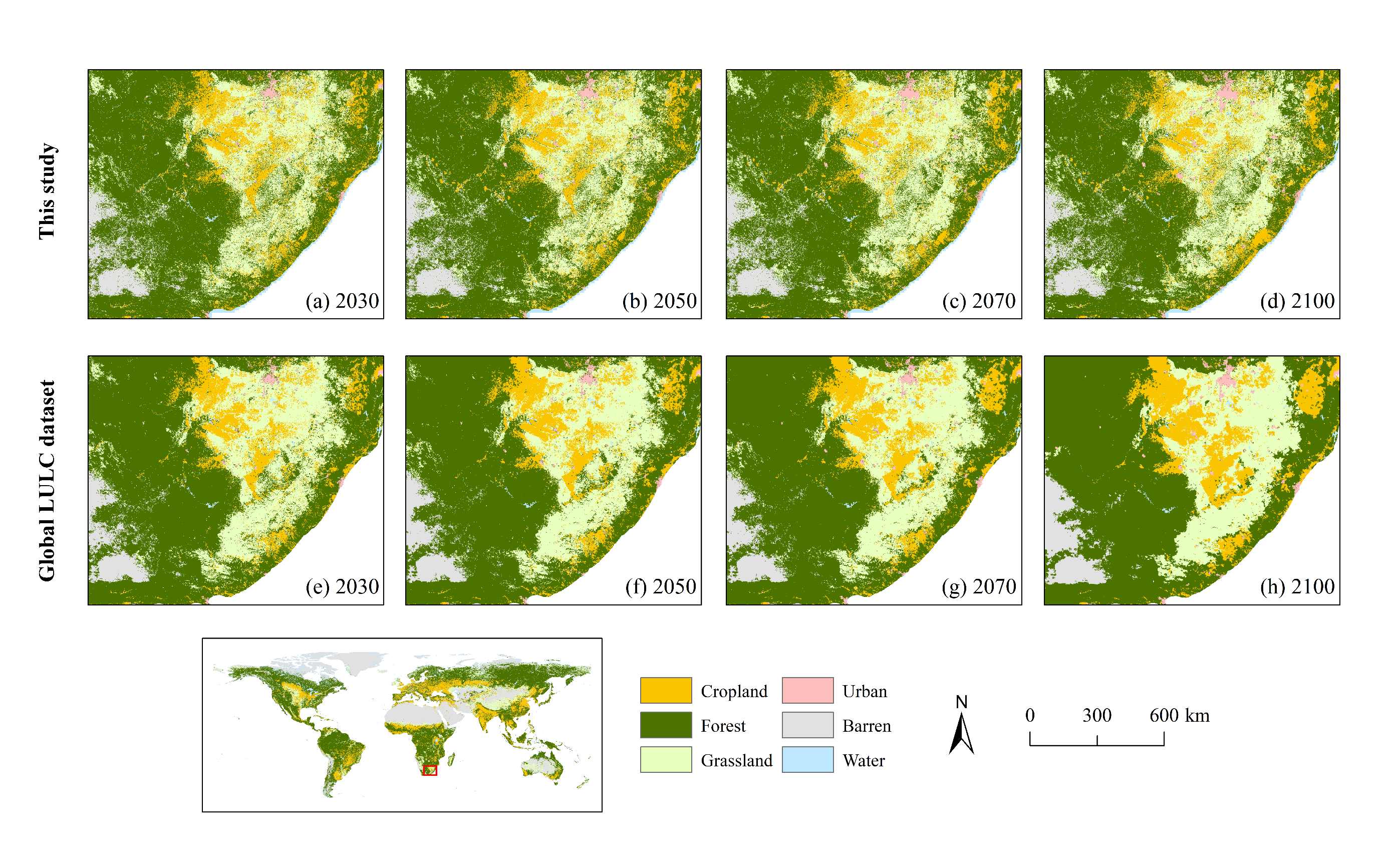


Figure S18. Comparison of the simulated LULC results of a typical case region (South Africa) in the years 2030, 2050, 2070 and 2100 under the SSP2-4.5 scenario between the dataset produced in this study and the *Global LULC dataset*^4^.

### Tables

Table S1. LULC classification and mapping relationships among different products. The table compares and maps the LULC types utilized in this study, ESA-CCI land cover data, LUH2^5^ and the *Global LULC dataset*^4^.

| **This Study** | **GCAM** | **ESA-CCI** | **LUH2** | **Global LULC dataset** |
| --- | --- | --- | --- | --- |
| Cropland | Cropland | Cropland, rainfed | C3 annual crops | Cropland |
|  |  | Cropland, irrigated or post-flooding | C4 annual crops |  |
|  |  | Mosaic cropland (>50%)/natural vegetation (tree, shrub, herbaceous cover) (<50%) | C3 perennial crops |  |
|  |  |  | C4 perennial crops |  |
|  |  |  | C3 nitrogen-fixing crops |  |
| Forest | Forest | Tree or shrub cover | Primary forest | Forest |
|  | Shrubland | Mosaic natural vegetation (tree, shrub, herbaceous cover) (>50%)/cropland (<50%) | Secondary forest |  |
|  |  | Tree cover, broad-leaved, evergreen, closed to open (>15%) |  |  |
|  |  | Tree cover, broad-leaved, deciduous, closed to open (>15%) |  |  |
|  |  | Tree cover, broad-leaved, deciduous, closed (>40%) |  |  |
|  |  | Tree cover, broad-leaved, deciduous, open (15–40%6) |  |  |
|  |  | Tree cover, needle-leaved, evergreen, closed to open (>15%) |  |  |
|  |  | Tree cover, needle-leaved, evergreen, closed (>40%) |  |  |
|  |  | Tree cover, needle-leaved, evergreen, open (15–40%) |  |  |
|  |  | Tree cover, needle-leaved, deciduous, closed to open (>15%) |  |  |
|  |  | Tree cover, needle-leaved, deciduous, closed (>40%) |  |  |
|  |  | Tree cover, needle-leaved, deciduous, open (15–40%) |  |  |
|  |  | Tree cover, mixed leaf type (broadleaved and needle-leaved) |  |  |
|  |  | Mosaic tree and shrub (>50%)/herbaceous cover (<50%) |  |  |
|  |  | Shrubland |  |  |
|  |  | Evergreen shrubland |  |  |
|  |  | Deciduous shrubland |  |  |
|  |  | Tree cover, flooded, fresh or brackish water |  |  |
|  |  | Tree cover, flooded, saline water |  |  |
| Grassland | Grassland | Grassland | Managed pasture | Grassland |
|  | Pasture | Herbaceous cover | Rangeland |  |
|  |  | Mosaic herbaceous cover (>50%)/tree and shrub (<50%) |  |  |
|  |  | Shrub or herbaceous cover, flooded, fresh/saline/brackish water |  |  |
| Water |  | Water bodies |  | Water |
| Urban | Urban land | Urban areas | Urban land | Urban |
| Barren | Tundra | Lichens and mosses | Primary non-forest | Barren |
|  | Rock/Ice/Desert | Sparse vegetation (tree, shrub. herbaceous cover) (<15%) | Secondary non-forest |  |
|  |  | Sparse shrub (<15%) |  |  |
|  |  | Sparse herbaceous cover (<15%) |  |  |
|  |  | Bare areas |  |  |
|  |  | Consolidated bare areas |  |  |
|  |  | Unconsolidated bare areas |  |  |
|  |  | Permanent snow and ice |  | Permanent snow and ice |

Table S2. The FoM, Kappa coefficient, and OA values for each water-basin region. The regions primarily situated within China are highlighted in red.

| **Basin code** | **FoM** | **Kappa** | **OA** |
| --- | --- | --- | --- |
| 1 | 0.06 | 0.97 | 0.98 |
| 2 | 0.11 | 0.99 | 1.00 |
| 3 | 0.08 | 0.97 | 0.98 |
| 4 | 0.06 | 0.93 | 0.96 |
| 5 | 0.09 | 0.95 | 0.96 |
| 6 | 0.06 | 0.88 | 0.98 |
| 7 | 0.02 | 0.97 | 0.98 |
| 8 | 0.13 | 0.91 | 0.94 |
| 9 | 0.06 | 0.92 | 0.96 |
| 10 | 0.12 | 0.94 | 0.97 |
| 11 | 0.06 | 0.97 | 0.98 |
| 12 | 0.12 | 0.90 | 0.94 |
| 13 | 0.10 | 0.88 | 0.94 |
| 14 | 0.08 | 0.87 | 0.97 |
| 15 | 0.08 | 0.94 | 0.96 |
| 16 | 0.05 | 0.97 | 0.98 |
| 17 | 0.07 | 0.95 | 0.98 |
| 18 | 0.10 | 0.94 | 0.96 |
| 19 | 0.05 | 0.97 | 0.98 |
| 20 | 0.15 | 0.94 | 0.96 |
| 21 | 0.07 | 0.96 | 0.97 |
| 22 | 0.06 | 0.95 | 0.97 |
| 23 | 0.08 | 0.96 | 0.98 |
| 24 | 0.09 | 0.96 | 0.98 |
| 25 | 0.04 | 0.93 | 0.96 |
| 26 | 0.07 | 0.93 | 0.97 |
| 27 | 0.13 | 0.85 | 0.95 |
| 28 | 0.11 | 0.92 | 0.95 |
| 29 | 0.06 | 0.96 | 0.97 |
| 30 | 0.09 | 0.95 | 0.97 |
| 31 | 0.07 | 0.93 | 0.95 |
| 32 | 0.08 | 0.92 | 0.95 |
| 33 | 0.06 | 0.95 | 0.96 |
| 34 | 0.02 | 0.96 | 0.98 |
| 35 | 0.05 | 0.94 | 0.96 |
| 36 | 0.09 | 0.82 | 0.90 |
| 37 | 0.13 | 0.90 | 0.97 |
| 38 | 0.09 | 0.92 | 0.94 |
| 39 | 0.08 | 0.92 | 0.96 |
| 40 | 0.08 | 0.87 | 0.96 |
| **Basin code** | **FoM** | **Kappa** | **OA** |
| 41 | 0.06 | 0.93 | 0.96 |
| 42 | 0.06 | 0.90 | 0.95 |
| 43 | 0.02 | 0.93 | 0.96 |
| 44 | 0.07 | 0.95 | 0.97 |
| 45 | 0.16 | 0.89 | 0.92 |
| 46 | 0.08 | 0.95 | 0.97 |
| 47 | 0.06 | 0.97 | 0.98 |
| 48 | 0.04 | 0.92 | 0.98 |
| 49 | 0.08 | 0.92 | 0.95 |
| 50 | 0.14 | 0.96 | 0.97 |
| 51 | 0.15 | 0.93 | 0.96 |
| 52 | 0.08 | 0.97 | 0.99 |
| 53 | 0.07 | 0.96 | 0.98 |
| 54 | 0.04 | 0.94 | 0.96 |
| 55 | 0.08 | 0.97 | 0.98 |
| 56 | 0.10 | 0.92 | 0.95 |
| 57 | 0.13 | 0.94 | 0.96 |
| 58 | 0.51 | 0.97 | 0.98 |
| 59 | 0.17 | 0.96 | 0.97 |
| 60 | 0.08 | 0.91 | 0.95 |
| 61 | 0.14 | 0.91 | 0.95 |
| 62 | 0.07 | 0.94 | 0.96 |
| 63 | 0.12 | 0.98 | 0.98 |
| 64 | 0.07 | 0.96 | 0.97 |
| 65 | 0.17 | 0.91 | 0.94 |
| 66 | 0.15 | 0.91 | 0.93 |
| 67 | 0.12 | 0.96 | 0.97 |
| 68 | 0.08 | 0.86 | 0.90 |
| 69 | 0.13 | 0.97 | 0.98 |
| 70 | 0.09 | 0.96 | 0.98 |
| 71 | 0.15 | 0.96 | 0.97 |
| 72 | 0.24 | 0.94 | 0.96 |
| 73 | 0.07 | 0.98 | 0.98 |
| 74 | 0.09 | 0.98 | 0.99 |
| 75 | 0.12 | 0.97 | 0.97 |
| 76 | 0.08 | 0.84 | 0.89 |
| 77 | 0.14 | 0.87 | 0.91 |
| 78 | 0.22 | 0.88 | 0.92 |
| 79 | 0.06 | 0.97 | 0.98 |
| 80 | 0.31 | 0.95 | 0.97 |
| **Basin code** | **FoM** | **Kappa** | **OA** |
| 81 | 0.05 | 0.98 | 0.99 |
| 82 | 0.17 | 0.92 | 0.98 |
| 83 | 0.06 | 0.97 | 0.98 |
| 84 | 0.14 | 0.96 | 0.98 |
| 85 | 0.07 | 0.92 | 0.96 |
| 86 | 0.07 | 0.97 | 0.99 |
| 87 | 0.03 | 0.96 | 0.97 |
| 88 | 0.14 | 0.96 | 0.99 |
| 89 | 0.17 | 0.94 | 0.96 |
| 90 | 0.08 | 0.93 | 0.96 |
| 91 | 0.03 | 0.99 | 0.99 |
| 92 | 0.02 | 0.89 | 0.96 |
| 93 | 0.12 | 0.97 | 0.99 |
| 94 | 0.13 | 0.96 | 1.00 |
| 95 | 0.16 | 0.96 | 0.97 |
| 96 | 0.09 | 0.94 | 0.98 |
| 97 | 0.07 | 0.92 | 0.96 |
| 98 | 0.16 | 0.99 | 1.00 |
| 99 | 0.23 | 0.95 | 0.99 |
| 100 | 0.05 | 0.96 | 0.97 |
| 101 | 0.07 | 0.97 | 0.98 |
| 102 | 0.07 | 0.85 | 0.96 |
| 103 | 0.15 | 0.90 | 0.94 |
| 104 | 0.20 | 0.87 | 0.94 |
| 105 | 0.03 | 0.98 | 0.98 |
| 106 | 0.12 | 0.94 | 0.96 |
| 107 | 0.13 | 0.97 | 0.98 |
| 108 | 0.14 | 0.90 | 0.95 |
| 109 | 0.07 | 0.94 | 0.97 |
| 110 | 0.10 | 0.97 | 0.98 |
| 111 | 0.05 | 0.95 | 0.98 |
| 112 | 0.11 | 0.94 | 0.96 |
| 113 | 0.08 | 0.93 | 0.98 |
| 114 | 0.08 | 0.95 | 0.98 |
| 115 | 0.05 | 0.91 | 0.97 |
| 116 | 0.10 | 0.97 | 0.99 |
| 117 | 0.11 | 0.93 | 0.97 |
| 118 | 0.18 | 0.94 | 0.98 |
| 119 | 0.06 | 0.97 | 0.99 |
| 120 | 0.07 | 0.97 | 0.98 |
| 122 | 0.07 | 0.95 | 0.98 |
| 123 | 0.09 | 0.96 | 0.98 |
| **Basin code** | **FoM** | **Kappa** | **OA** |
| 124 | 0.15 | 0.97 | 0.98 |
| 125 | 0.11 | 0.95 | 0.97 |
| 126 | 0.08 | 0.95 | 0.97 |
| 127 | 0.02 | 0.98 | 0.99 |
| 128 | 0.08 | 0.96 | 0.98 |
| 129 | 0.13 | 0.94 | 0.96 |
| 130 | 0.07 | 0.96 | 0.98 |
| 131 | 0.08 | 0.91 | 0.95 |
| 132 | 0.15 | 0.96 | 0.98 |
| 133 | 0.09 | 0.93 | 0.97 |
| 134 | 0.06 | 0.95 | 0.98 |
| 135 | 0.09 | 0.96 | 0.98 |
| 136 | 0.05 | 0.97 | 0.98 |
| 137 | 0.02 | 0.96 | 0.98 |
| 138 | 0.05 | 0.95 | 0.97 |
| 139 | 0.11 | 0.93 | 0.96 |
| 140 | 0.09 | 0.92 | 0.97 |
| 142 | 0.13 | 0.93 | 0.96 |
| 143 | 0.06 | 0.96 | 0.98 |
| 144 | 0.11 | 0.91 | 1.00 |
| 145 | 0.15 | 0.90 | 0.96 |
| 146 | 0.14 | 0.96 | 0.98 |
| 147 | 0.05 | 0.97 | 0.99 |
| 148 | 0.10 | 0.94 | 0.96 |
| 149 | 0.11 | 0.94 | 0.96 |
| 151 | 0.08 | 0.97 | 0.98 |
| 152 | 0.06 | 0.94 | 0.96 |
| 153 | 0.14 | 0.96 | 0.98 |
| 154 | 0.05 | 0.94 | 0.96 |
| 155 | 0.12 | 0.97 | 0.98 |
| 156 | 0.04 | 0.97 | 0.98 |
| 157 | 0.13 | 0.93 | 0.97 |
| 158 | 0.06 | 0.97 | 0.99 |
| 159 | 0.12 | 0.78 | 0.92 |
| 160 | 0.09 | 0.93 | 0.96 |
| 161 | 0.05 | 0.94 | 0.99 |
| 162 | 0.05 | 0.93 | 0.98 |
| 163 | 0.12 | 0.87 | 0.93 |
| 164 | 0.07 | 0.94 | 0.96 |
| 165 | 0.11 | 0.83 | 0.93 |
| 166 | 0.08 | 0.93 | 0.97 |
| 167 | 0.02 | 0.94 | 0.98 |
| **Basin code** | **FoM** | **Kappa** | **OA** |
| 168 | 0.11 | 0.85 | 0.97 |
| 169 | 0.07 | 0.98 | 0.99 |
| 170 | 0.07 | 0.95 | 0.98 |
| 171 | 0.04 | 0.95 | 0.98 |
| 172 | 0.01 | 0.89 | 0.94 |
| 173 | 0.06 | 0.97 | 0.98 |
| 174 | 0.13 | 0.89 | 0.94 |
| 175 | 0.13 | 0.94 | 0.95 |
| 176 | 0.01 | 0.91 | 1.00 |
| 177 | 0.05 | 0.95 | 0.97 |
| 178 | 0.06 | 0.97 | 0.98 |
| 179 | 0.07 | 0.95 | 0.97 |
| 180 | 0.05 | 0.96 | 0.97 |
| 181 | 0.16 | 0.81 | 0.97 |
| 182 | 0.09 | 0.93 | 0.97 |
| 183 | 0.06 | 0.92 | 0.98 |
| 184 | 0.06 | 0.97 | 0.98 |
| 185 | 0.15 | 0.73 | 0.84 |
| 186 | 0.05 | 0.94 | 0.97 |
| 187 | 0.02 | 0.88 | 0.96 |
| 188 | 0.02 | 0.96 | 0.98 |
| 189 | 0.07 | 0.97 | 0.98 |
| 190 | 0.07 | 0.94 | 0.97 |
| 191 | 0.04 | 0.93 | 0.95 |
| 192 | 0.08 | 0.95 | 0.97 |
| 193 | 0.09 | 0.85 | 0.92 |
| 194 | 0.06 | 0.97 | 0.98 |
| 195 | 0.06 | 0.94 | 0.96 |
| 196 | 0.23 | 0.92 | 0.96 |
| 197 | 0.07 | 0.96 | 0.98 |
| 198 | 0.11 | 0.95 | 0.97 |
| 199 | 0.10 | 0.94 | 0.98 |
| 200 | 0.07 | 0.95 | 0.98 |
| 201 | 0.02 | 0.96 | 0.97 |
| **Basin code** | **FoM** | **Kappa** | **OA** |
| 202 | 0.11 | 0.91 | 0.94 |
| 203 | 0.19 | 0.91 | 0.95 |
| 204 | 0.20 | 0.98 | 0.99 |
| 205 | 0.06 | 0.97 | 0.98 |
| 206 | 0.07 | 0.93 | 0.96 |
| 207 | 0.13 | 0.98 | 0.99 |
| 208 | 0.07 | 0.94 | 0.96 |
| 209 | 0.02 | 0.93 | 0.95 |
| 210 | 0.17 | 0.98 | 0.99 |
| 211 | 0.29 | 0.98 | 0.99 |
| 212 | 0.07 | 0.98 | 0.99 |
| 213 | 0.15 | 0.99 | 0.99 |
| 214 | 0.29 | 0.99 | 0.99 |
| 215 | 0.12 | 0.98 | 0.99 |
| 217 | 0.13 | 0.96 | 0.98 |
| 218 | 0.13 | 0.94 | 0.97 |
| 219 | 0.05 | 0.95 | 0.97 |
| 220 | 0.03 | 0.96 | 0.99 |
| 221 | 0.33 | 0.92 | 0.99 |
| 222 | 0.14 | 0.95 | 0.99 |
| 223 | 0.05 | 0.97 | 0.99 |
| 224 | 0.04 | 0.95 | 0.98 |
| 225 | 0.16 | 0.96 | 0.97 |
| 226 | 0.09 | 0.90 | 0.95 |
| 227 | 0.28 | 0.97 | 0.98 |
| 228 | 0.11 | 0.95 | 0.97 |
| 229 | 0.10 | 0.95 | 0.98 |
| 230 | 0.05 | 0.89 | 0.94 |
| 231 | 0.15 | 0.95 | 0.98 |
| 232 | 0.17 | 0.92 | 0.98 |
| 233 | 0.14 | 0.94 | 0.97 |
| 234 | 0.23 | 0.98 | 0.99 |
| 235 | 0.04 | 0.92 | 0.96 |
| **Average** | 0.10 | 0.94 | 0.97 |

### References

1. Pontius, R. G. *et al.* Comparing the input, output, and validation maps for several models of land change. *Ann. Reg. Sci.* **42**, 11–37 (2008).

2. Pontius, R. G. & Millones, M. Death to Kappa: birth of quantity disagreement and allocation disagreement for accuracy assessment. *International Journal of Remote Sensing* **32**, 4407–4429 (2011).

3. Liu, X. *et al.* A future land use simulation model (FLUS) for simulating multiple land use scenarios by coupling human and natural effects. *Landscape and Urban Planning* **168**, 94–116 (2017).

4. Chen, G., Li, X. & Liu, X. Global land projection based on plant functional types with a 1-km resolution under socio-climatic scenarios. *Sci Data* **9**, 125 (2022).

5. Hurtt, G. C. *et al.* Harmonization of global land use change and management for the period 850–2100 (LUH2) for CMIP6. *Geoscientific Model Development* **13**, 5425–5464 (2020).
